# Observed and Modeled Black Carbon Deposition and Sources in the Western Russian Arctic 1800–2014

**DOI:** 10.1021/acs.est.0c07656

**Published:** 2021-03-26

**Authors:** Meri M. Ruppel, Sabine Eckhardt, Antto Pesonen, Kenichiro Mizohata, Markku J. Oinonen, Andreas Stohl, August Andersson, Vivienne Jones, Sirkku Manninen, Örjan Gustafsson

**Affiliations:** † Ecosystems and Environment Research Programme, Faculty of Biological and Environmental Sciences, University of Helsinki, FI-00014 Helsinki, Finland; ‡ Norwegian Institute for Air Research (NILU), NO-2027 Kjeller, Norway; § Technology Center, Neste Corporation, FI-06101 Porvoo, Finland; ∥ Laboratory of Chronology, Finnish Museum of Natural History—LUOMUS, University of Helsinki, FI-00014 Helsinki, Finland; # Department of Meteorology and Geophysics, University of Vienna, A-1090 Vienna, Austria; ¶ Department of Environmental Science and the Bolin Centre for Climate Research, Stockholm University, SE-106 91 Stockholm, Sweden; ∇ Environmental Change Research Centre, Department of Geography, University College London, WC1E 6BT London, U.K.; ⊥ Division of Materials Physics, Department of Physics, University of Helsinki, FI-00014 Helsinki, Finland

## Abstract

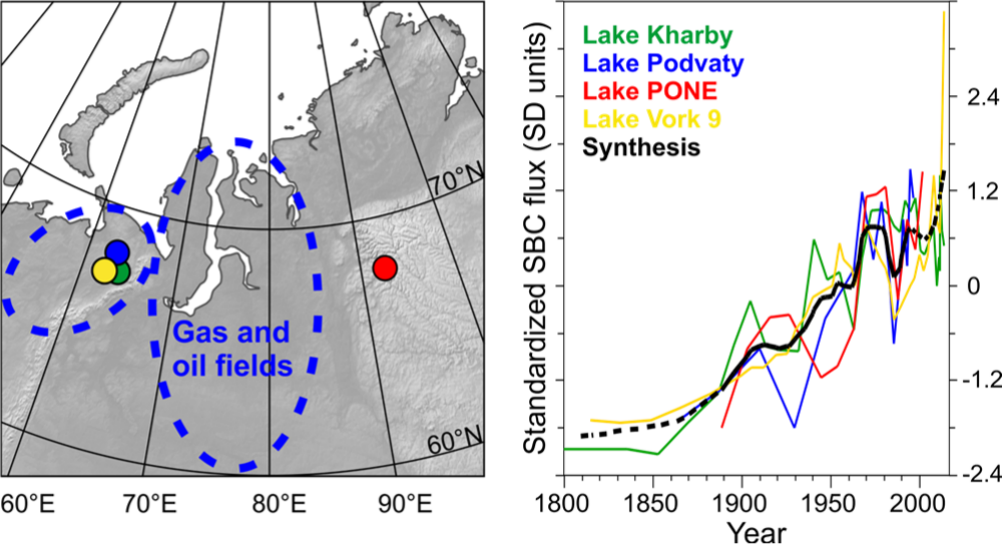

Black carbon (BC) particles contribute to climate warming by heating the atmosphere and reducing the albedo of snow/ice surfaces. The available Arctic BC deposition records are restricted to the Atlantic and North American sectors, for which previous studies suggest considerable spatial differences in trends. Here, we present first long-term BC deposition and radiocarbon-based source apportionment data from Russia using four lake sediment records from western Arctic Russia, a region influenced by BC emissions from oil and gas production. The records consistently indicate increasing BC fluxes between 1800 and 2014. The radiocarbon analyses suggest mainly (∼70%) biomass sources for BC with fossil fuel contributions peaking around 1960–1990. Backward calculations with the atmospheric transport model FLEXPART show emission source areas and indicate that modeled BC deposition between 1900 and 1999 is largely driven by emission trends. Comparison of observed and modeled data suggests the need to update anthropogenic BC emission inventories for Russia, as these seem to underestimate Russian BC emissions and since 1980s potentially inaccurately portray their trend. Additionally, the observations may indicate underestimation of wildfire emissions in inventories. Reliable information on BC deposition trends and sources is essential for design of efficient and effective policies to limit climate warming.

## 1 Introduction

Black carbon (BC) particulates are produced by incomplete combustion of carbonaceous material both naturally (e.g., from forest fires) and in anthropogenic activities (e.g., energy production, residential burning, industry, and traffic). BC has an atmospheric lifetime of a few days to weeks during which it may be transported over thousands of kilometers before wet or dry deposition processes remove it from the atmosphere.^1^ BC has the strongest light absorption property of all particulates and is considered the second or third most important global climate warming agent after carbon dioxide and perhaps methane but with large uncertainties in radiative forcing.^2^

The climatic effect of BC is amplified in the Arctic where its deposition on snow and ice decreases their reflectivity and hastens melt due to within-snow feedback processes related to the BC-driven increased heat absorption. Furthermore, the light absorption of atmospheric BC is enhanced over high-reflectivity surfaces.^2,3^ One quarter of the present warming in the Arctic may be caused by BC.^2^

Observational BC data are mostly available from atmospheric monitoring showing decreasing atmospheric BC concentrations throughout the Arctic between 1990 and 2009.^2,4^ However, field data on Arctic BC deposition and particularly its sources are scarce. Long-term data have been obtained mostly from Greenland ice cores, representing BC deposition only at these high-elevation sites receiving emissions mostly from North America, showing decreasing or stable BC trends in recent decades.^5,6^ However, some European Arctic ice core and lake sediment data show increasing BC deposition trends since the 1970s^7,8^ and suggest spatially variable BC trends.^9^ Observational data providing constraints on BC sources for the Arctic are mainly available for atmospheric,^10,11^ snow,^12,13^ and estuarine surface sediments,^14^ which represent snapshots in time. While BC sources and particularly their trends are poorly constrained in most of the Arctic, atmospheric isotopic BC source analyses suggest that the spatial allocation of emissions and their source contributions in emission inventory-based models may need significant improvement.^10^ Reliable information on BC trends and sources is essential to evaluate the climatic impact of BC in the past, present, and future, as well as to correctly target BC mitigation efforts.

Models suggest that emissions from Asia contribute most to the Arctic BC burden and that per unit emitted mass, the Arctic surface temperature is most sensitive to Russian flaring emissions.^2,15^ Until recently, BC emissions from natural gas flaring have been significantly underestimated or even disregarded in emission inventories.^16^ While flaring causes globally only ca. 3% of total BC emissions, it is a significant BC emission source within the Arctic and has been estimated to cause as much as 42% of annual average surface BC concentrations.^16^ According to satellite data, Russia is and has been by far the leading country in flaring with activity increasing from 1994 to ca. 2005 and decreasing since then,^17,18^ which is now incorporated in updated emission inventories.^19^

Thus, flaring may have a pronounced impact on Arctic climate, but very few studies on its environmental effect exist. Here, we present first long-term observational data on BC deposition in Russia accompanied by first-of-a-kind Arctic radiocarbon source data over the last ca. 200 years. We use four lake sediment records, preserving chemically inert BC in chronological succession, located in close proximity to the most intense Russian flaring areas. This information will help evaluate the potential importance of flaring as an Arctic BC source in the past and present and will enlighten how Arctic industrial development may affect future trends of Arctic BC deposition.

## 2 Materials and Methods

### 2.1 Study Area

**Figure 1:**
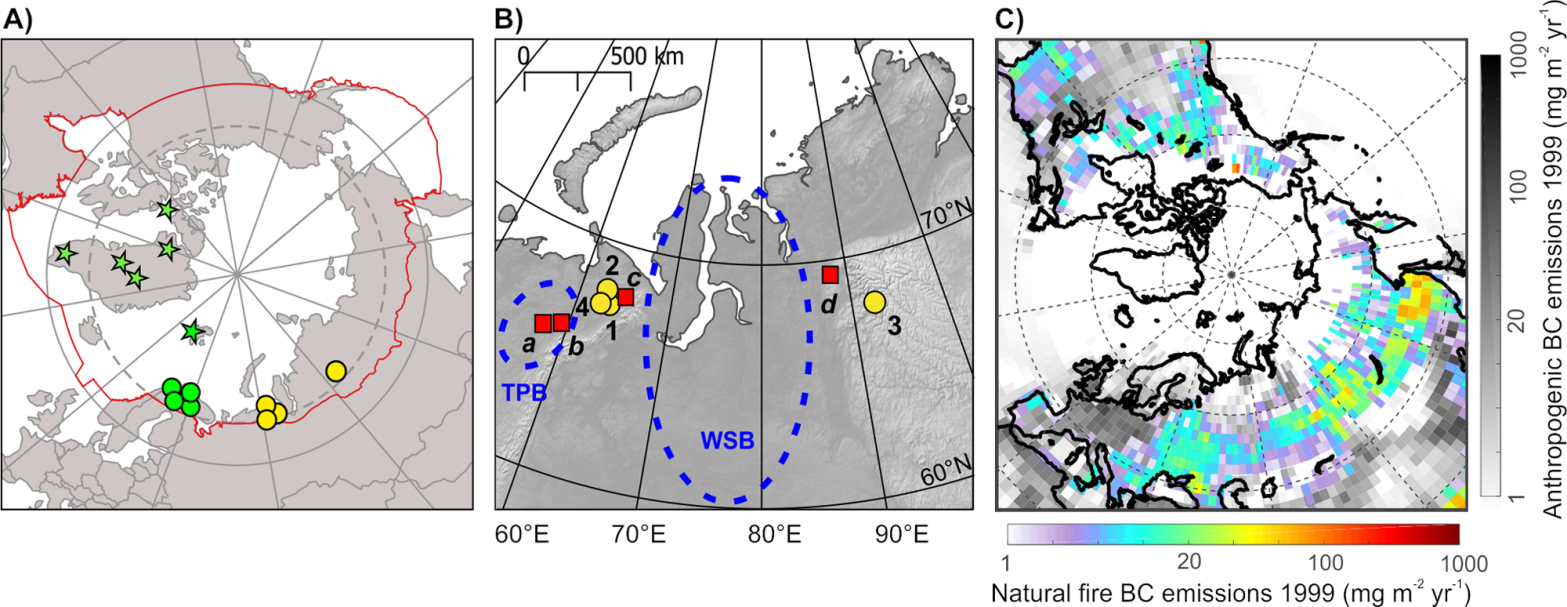
Study sites and BC emissions in their vicinity. (A) Locations of the study lakes (yellow dots), previously published lake sediment BC records from northern Finland (green dots),^8^ and Svalbard,^7,9,46^ Greenland,^56,60^ and a Canadian^61^ ice core records (green stars). The red line indicates the Arctic as defined by the Arctic Monitoring and Assessment Programme (AMAP). (B) Location of the study lakes (yellow dots: 1 = Kharby; 2 = Podvaty; 3 = PONE; and 4 = Vork 9) in the vicinity of industrial cities (red squares: a = Usinsk; b = Inta; c = Vorkuta; and d = Norilsk). The blue dashed lines show the approximate location of flaring activity in the Timan–Pechora Basin (TPB) and the West Siberian Basin (WSB). (C) CMIP6 anthropogenic BC emissions for 1999^19^ (gray log scale) and fire BC emissions of open fires for 1999^43^ (color log scale) in mg m^–2^ yr^–1^.

The bulk of flaring in Russia occurs in two areas: the Timan–Pechora Basin and the West Siberian Basin with massive gas and oil reserves (Figure 1). Drilling activity increased dramatically in the Timan–Pechora Basin oil and gas fields in the mid-1940s and has continued to the present.^20^ Additionally, extensive coal mining started in the area in the 1940s.^21^ Large-scale production in the West Siberian Basin started in the early 1970s, and it produces today over three quarters of Russian gas and oil.^22^ As flaring is a byproduct of gas and oil extraction, more specifically a “disposal process of dissolved natural gas present in petroleum in production and processing facilities where there is no infrastructure to make use of the gas”,^17^ it can be assumed that flaring developed in parallel to the drilling activities. By the 1970s, night-time infrared satellite images detected flaring in Russia and around the world at known oil and gas fields.^23^

The studied lakes (Table S1) Kharby, Podvaty, and Vork 9 are located within a 20 km radius from one another, approximately 50 km west of the largest industrial coal mining center (Vorkuta) and 300 km northeast of the largest gas flaring center (Usinsk) in the Timan–Pechora Basin (Figure 1). These lakes were chosen to study the influence of within-Arctic industrial development on BC deposition and to test the repeatability of the results from different types of lakes in the same area. The study lake PONE, located ca. 1000 km east of the other study lakes and ca. 400 km east of the closest West Siberian Basin oil and gas fields, was chosen to study BC deposition patterns on a wider geographical scale together with the other lakes (Figure 1).

### 2.2 Sediment Collection and Radiometric Dating

The lake sediments were collected between 1998 and 2014 for various environmental studies from the Timan–Pechora Basin (Kharby, Podvaty, and Vork 9) and the Putorana Plateau (PONE) (Figure 1) by researchers of University College London.^21,24^ Lakes with simple bathymetry, small catchment sizes, and no or small in- and outflow systems were favored, so that the relative contribution of the material deposited directly from the atmosphere, rather than as secondary influx from the catchment area or the sediment bed, was kept as high as possible. More sample collection details are given in the Supporting Information (S.1.).

For radiometric dating, the sediments were analyzed for ^210^Pb, ^226^Ra, ^137^Cs, and ^241^Am by direct gamma assay at the University College London. Details on the dating methodology are given elsewhere^25−27^ and the results in Tables S2–S5. Dating uncertainties are around 2–10 years for the 20th century and increase toward older sediment layers, with a 1–2 year resolution at the top of the cores and a decrease in depth.

### 2.3 Soot BC Quantification

BC was quantified using the chemo-thermal oxidation method at 375 °C (CTO-375) which was developed to quantify BC from sediments^28,29^ and atmospheric samples.^30^ This method effectively quantifies the most condensed high-refractory fraction of BC, the so-called soot BC (SBC), formed in high-temperature combustion flames of both bio- and fossil fuels.^31^ SBC measurements represent a subset of total BC as less condensed forms of char-BC are not quantified.^31^

The CTO-375 method is described in detail with evaluation of method accuracy and precision in Supporting Information Sections S.3.–S.4. In brief, after drying and homogenizing, the sediments were subjected to oxidation at 375 °C to remove organic material, followed by acidification (1 M HCl) to remove carbonates, and subsequently, the remaining carbon left in the samples quantified as SBC with an elemental analyzer (Thermo Scientific Flash 2000 NC).

SBC concentrations are reported in Figure S1. SBC fluxes (SBC deposition to the sediments) were calculated based on the measured SBC concentrations and the sedimentation rate in the cores (reported in the Supporting Information). The calculation was constrained by the radiometric dating using excess ^210^Pb (half-life of 22.3 years), and thus, SBC fluxes are presented only for the last ca. 120–160 years (Tables S2–S5).

### 2.4 Radiocarbon Source Apportionment

Major BC emission source categories can be identified by analyzing the radiocarbon (^14^C) content of SBC.^10,14,30^ The radiocarbon signature specifies the fraction of contemporary biomass versus fossil fuel (devoid of radiocarbon) combustion sources of SBC particles. This distinction is important, for instance, as the required mitigation measures to reduce BC emissions from the different sources vary substantially.

Here, 16 samples from Vork 9 were selected for radiocarbon source apportionment analyses. Vork 9 was the only sediment core where SBC concentrations were sufficient for accurate radiocarbon analyses. The samples were prepared for the radiocarbon analysis with the same SBC isolation steps as described above (Section 2.3). During the final step of SBC combustion in the elemental analyzer, CO_2_ evolved directly from the sediment SBC was cryogenically trapped. Two to three sediment samples of 10 mg size were pooled together into one trap to extract enough CO_2_ (ca. 20 μg of C) for the radiocarbon analyses.

The radiocarbon analyses were performed on the gaseous CO_2_ samples by accelerator mass spectrometry (AMS) using a gas injection system for the 40 cathode SNICS (Source of Negative Ions by Cesium Sputtering) hybrid ion source. The hybrid ion source instrumentation is described in more detail in the Supporting Information (Section S.6.), and the results are compared to AMS measurements prepared by traditional preparation steps including graphitization (Sections S.7. and S.9.).^32,33^

### 2.5 Modeling

To explore the emission source areas and historical BC deposition in the study area, the atmospheric transport model FLEXPART (Flexible Particle Dispersion Model)^34−36^ was used for the period 1900–1999. The model was driven with the coupled climate reanalysis for the 20th century^37^ produced at the European Centre for Medium Range Weather Forecasts (ECMWF), used here at a resolution of 2° × 2° and 91 vertical levels and every 6 h. FLEXPART is widely used for establishing source–receptor relationships and has been shown to capture well BC transport to the Arctic,^16,38−40^ in addition to Arctic atmospheric BC concentrations and their seasonality.^10,11,41^ Model uncertainties are discussed in Supporting Information S.10.

Here, the source–receptor relationships for deposited quantities were calculated in backward mode.^35^ This method is an extension of similar methods developed for atmospheric concentrations and allows efficient calculation of high-resolution emission sensitivities for substances recorded in deposition records.^42^ Monthly backward simulations, releasing particles continuously over the months, were performed from the two study areas (Vork 9 representing all lakes in the Timan–Pechora Basin, and PONE) over the period 1900–1999. Each simulation traced BC particles 30 days backward in time from the lake sediment site and the time of deposition. Separate simulations were performed for dry and wet deposition using 100,000 and 500,000 particles, respectively. The model output is a gridded sensitivity of deposition to emissions with a resolution of 2° × 2°. The lowest atmospheric layer extends from the ground to 100 m above and is particularly relevant since most emissions occur near the surface. Multiplying the emission sensitivity for this layer with BC emission fluxes gives a source contribution map, and area integration of the source contributions gives the simulated monthly BC deposition. For this, historical CMIP6 (Coupled Model Intercomparison Project Phase 6) anthropogenic emissions^19^ and biomass burning emissions^43^ were used.

## 3 Results

### 3.1 Observed SBC Fluxes

**Figure 2:**
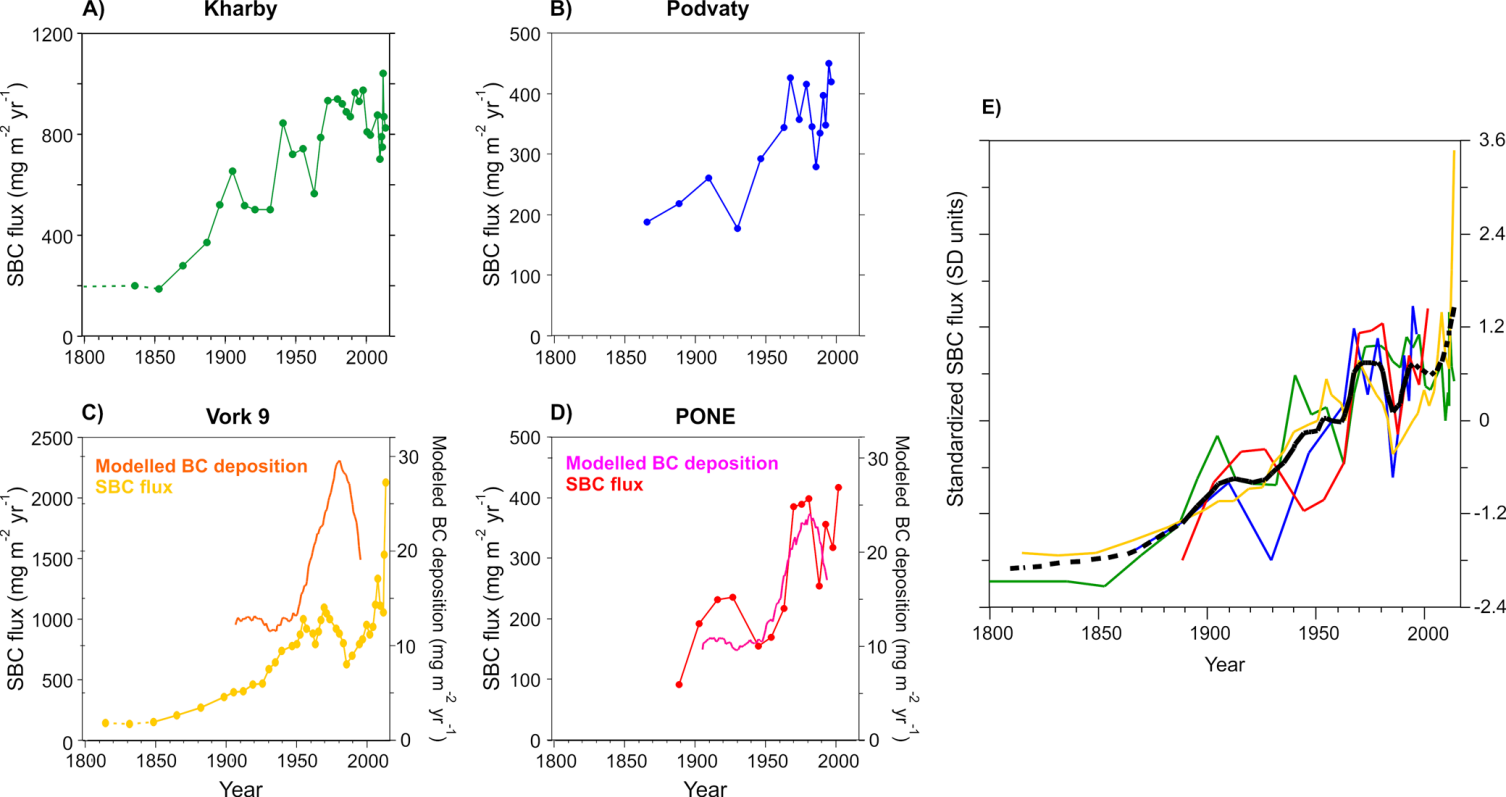
Sedimentary and standardized SBC fluxes observed in the Russian study lakes from 1800 to 2014 and modeled BC deposition between 1900 and 1999. (A–D) SBC fluxes (mg m^–2^ yr^–1^) in the sediment records. Note different *y*-axes between the lakes. Observations in Kharby and Vork 9 connected by dashed lines are from sediment sequences that have not been dated, and their age is estimated based on the sedimentation rate of the last dated sample. (C,D) Additionally, the modeled BC deposition to Vork 9 and PONE is shown on their own *y*-axes (mg m^–2^ yr^–1^) as 10-year running averages from 1900 to 1999. (E) Stacked SBC fluxes at Kharby, Podvaty, Vork 9, and PONE expressed as standard deviations from the mean. The black curve indicates a LOESS smoother (span 0.15) fitted to all records shown as dashed lines for time periods for which data are available only for some of the lakes.

The observed depositional fluxes of SBC show the temporal trend of atmospheric BC deposition to the sediment cores. The observed SBC fluxes in the four Russian lakes vary between ∼100 and 2200 mg m^–2^ yr^–1^ (Figure 2). The SBC fluxes in the Kharby and Vork 9 cores are almost twice as high as in Podvaty and PONE. This is presumably caused by these sediment cores receiving some re-suspended sediment input from other parts of the lake sediment bed and the catchment area, which is also suggested by their ^210^Pb fluxes that are twice as high as in Podvaty and PONE (Tables S2–S5). The radiometric dating of all cores shows that the cores reliably and consistently register material influx and thus SBC fluxes from the environment. However, due to possible material influx from the catchment area or the sediment bed to the coring locations, the exact atmospheric SBC deposition cannot be inferred from the sediment SBC fluxes presented in Figure 2, as discussed in ref (8). Moreover, individual SBC flux trends could be affected by external factors, such as delayed SBC export from the catchment area or dating biases. Consequently, the recurring features of the SBC flux trends in more than one lake (Figure 2e) are considered robust and representative of atmospheric BC deposition trends.^8^

All four lake sediment records show similar temporal patterns in SBC fluxes which are synthesized as standardized fluxes in Figure 2e. Kharby and Vork 9 show relatively constant and low SBC fluxes from the bottom of the cores to ca. 1850 (Figure 2a,c). In the latter half of the 19th century and the early 1900s, all cores indicate moderately to rapidly increasing SBC fluxes. Kharby and Podvaty show a short-term minimum in SBC fluxes around 1930 and PONE around 1940 (Figure 2a,b,d). Considering the PONE record’s dating error of ca. 17 years around 1930 [compared to ca. 5 years in Kharby and Podvaty in the same period (Tables S2–S5)], all these records may signal the same minimum in SBC fluxes around 1930. All sediment records indicate a rapid SBC flux increase beginning in the 1930s and steepening around 1960 (Figure 2). The fluxes remain high between 1960 and 1980. With the exception of Kharby, the SBC flux records show a marked brief minimum around 1986. From about the year 1986, the SBC fluxes in Podvaty, PONE, and Vork 9 increase to peak values similar or higher than between 1960 to 1980, while in Kharby, the SBC fluxes drop slightly in the 1990s (Figure 2). All records show peak values in their respective top layers, Podvaty at the end of 1990s, PONE in 2000s, and Kharby and Vork 9 in 2010s. To summarize, in all study lakes, SBC fluxes have markedly increased from the 1800s to the end of the records with peak values reached around 1960 to 1980, followed by a sharp minimum around 1986, and similar or higher than 1960 to 1980 values recorded at the end of the sediment records (Figure 2).

### 3.2 Modeled BC Fluxes

The modeled BC deposition from 1900 to 1999 is between 7 and 21 mg m^–2^ yr^–1^ for PONE and between 10 and 28 mg m^–2^ yr^–1^ for Vork 9 (Figure 2c,d). The modeled BC deposition is 1–2 magnitudes lower than the observed SBC fluxes in the sediment records. However, as the sedimentary SBC fluxes do not represent absolute atmospheric BC deposition and may include some BC influx from the catchment area, and models are known to underestimate BC concentrations^44,45^ and particularly deposition^8,46^ in the Arctic, comparison of relative trends is better justifiable than comparing exact values between model and observations. The temporal trends of the observed SBC fluxes and modeled BC deposition correlate moderately for the years from which both data are available (PONE: *r* = 0.64, *p* = 0.02; Vork 9: *r* = 0.61, *p* = 0.0008), with deviations occurring particularly after ca. 1980.

The modeled BC deposition remains at quite constant moderate levels from 1900 to 1940 and increases rapidly and constantly from ca. 1945 to peak values reached around 1980. After ca. 1980, the modeled BC deposition decreases toward 1999, reaching similar values as modeled for the 1960s (Figure 2c,d).

### 3.3 Radiocarbon-Based Source Apportionment of SBC

**Figure 3:**
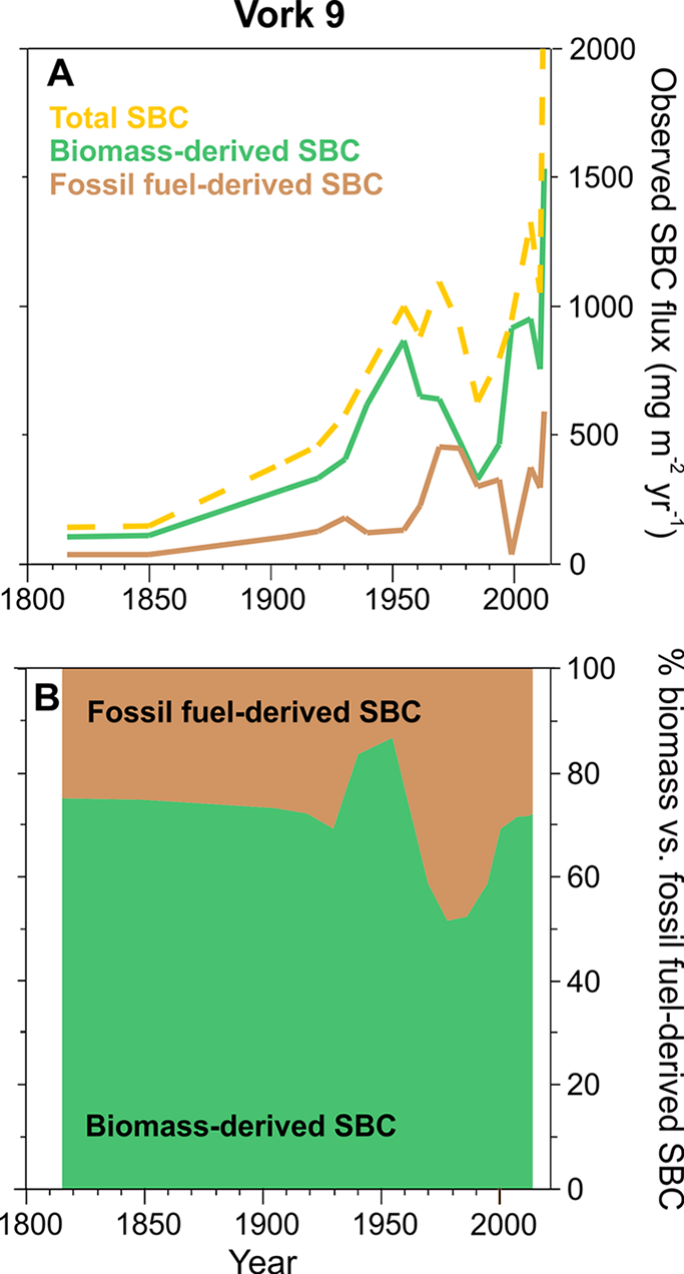
Contribution of biomass and fossil fuel-derived sources to the SBC extracted from the Vork 9 lake sediment sequence between 1800 and 2014 as temporal trends and percentages. (A) Temporal trend of SBC fluxes derived from biomass and fossil fuel sources. (B) Contribution (%) of biomass vs fossil fuel-derived sources to total SBC based on radiocarbon measurements of SBC. Errors of this data are typically between 1 and 2% (Table S7). Note that depending on the age of combusted peat, the emitted SBC may register in varying proportions as either or both biomass and fossil fuel-derived SBC.

Based on the radiocarbon analyses, throughout the studied period, on average 70% of the SBC deposited in the lake sediments was biomass derived (Figure 3). From the beginning of the 1800s to ca. 1950, about 75% of SBC originated from biomass combustion, while during the 1960s to 1990s, the contribution of fossil fuel sources to SBC increased to ca. 45%. In the 2000s, the contribution of biomass sources increased again to ca. 70% (Figure 3). We present first *long-term* radiocarbon sources of SBC in Russia and the Arctic, while previous studies have shown considerable spatial variation in Arctic BC sources with the contribution of biomass burning to BC ranging from 5 to 88% in pan-Arctic estuarine surface sediments^14^ and strong seasonal variation in the Arctic atmosphere with biomass sources dominating in summer and fossil sources in winter.^11^ When compared to other studies, it should be noted that SBC includes less fossil fuel-derived fractions of total BC than, for instance, elemental carbon^30^ due to its extraction methodology eliminating less condensed forms of BC such as coal (Supporting Information S.3.).^31^

### 3.4 Modeled Geographical BC Sources and Temporal Variation in Source Area Strengths

**Figure 4:**
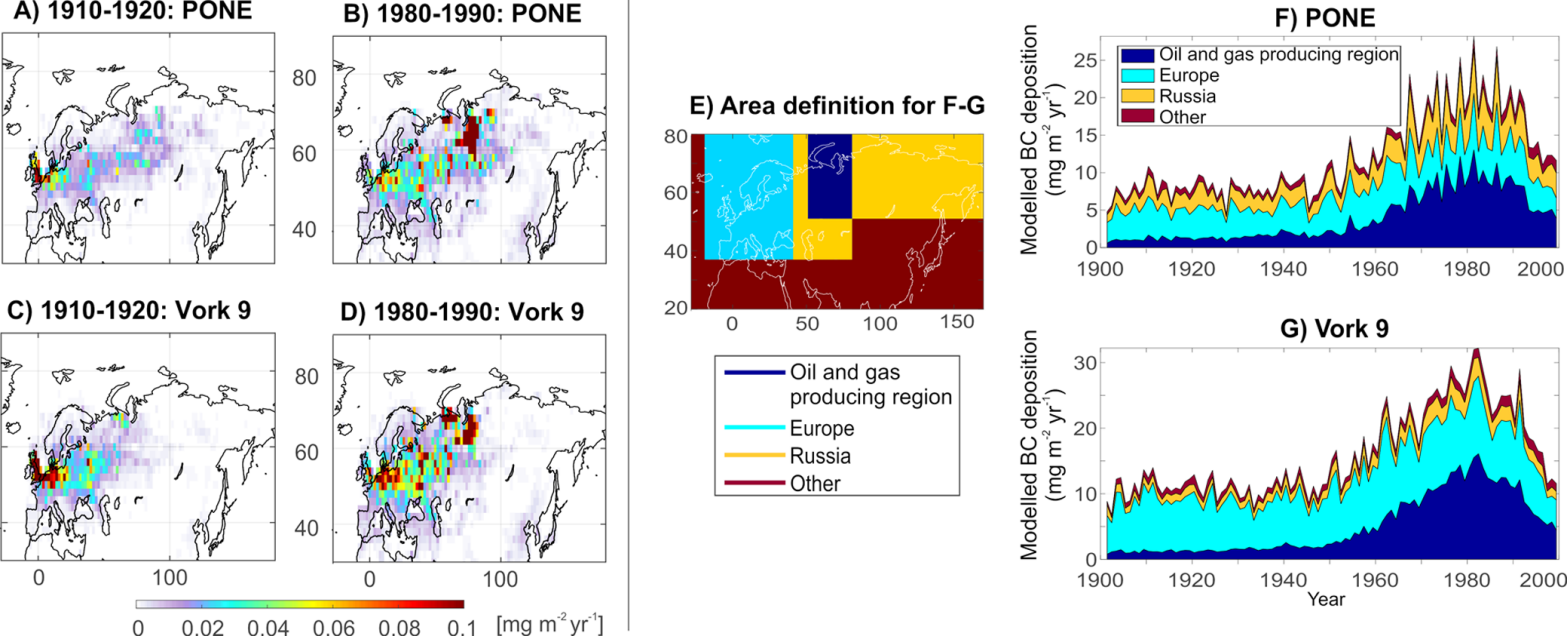
Source areas of BC deposited at the study lakes PONE and Vork 9 between 1900 and 1999. (A–D) 10 year averaged source contribution maps (mg m^–2^ yr^–1^) for PONE and Vork 9 for the period of 1910–1920 and 1980–1990. (E) Area definition for the panels (F,G). (F,G) Temporal variation of source contributions from the oil and gas producing region, the rest of Russia, Europe, and the rest of the world (other) sources in the 20th century.

According to the FLEXPART model results, the BC deposition in the study area is most sensitive to BC emissions in high-latitude Eurasia, particularly from Russian oil and gas fields and Northeastern Europe (Figure S3). Figure 4 shows source contribution maps for the periods 1910–1920 and 1980–1990. While in 1910–1920 the source contributions were dominated by European emissions, with comparably low contribution from emissions in the study region, by 1980–1990, the increased BC emissions from the oil and gas producing region (definition shown in Figure 4e) became most significant for the BC deposition at the study sites. Generally, the source contributions seem to have shifted eastward with time in the European part of the source area due to increasing emissions in Eastern Europe (Figure 4a–d). This shift in emission sources dominating the BC deposition at the study sites is also shown temporally in Figure 4f,g. While in 1910–1920 PONE and Vork 9 received ca. 16 and 11% of BC deposition from regional sources, that is, from the oil and gas producing region (Figure 4e), in 1980–1990, they received 45 and 49% of BC deposition from this area, respectively, with an accompanied decrease in the relative importance of European sources (Figure 4f,g). Generally, European emissions have been most significant for both study areas throughout the study period (ca. 36% for PONE and 51% for Vork 9), while emissions from other parts of Russia than the oil and gas producing region have influenced PONE more (ca. 24%) than Vork 9 (ca. 10%).

## 4 Discussion

### 4.1 Variation in Sources Explaining Observed SBC Deposition Trends

The SBC fluxes of the studied lakes are higher than previously recorded with the same analytical method in five Finnish Arctic lakes after ca. 1850^8^ even when considering their different sediment accumulation rates, indicating the influence of strong BC sources to the studied lakes. In a circum-Arctic snow BC survey, Russian snow packs were found to be the most polluted.^47^ Thus, BC emissions from the oil and gas producing area are relevant for SBC deposition in the study area, which may be one of the most BC polluted areas in the Arctic.

The observed sediment SBC deposition trends are remarkably consistent between the records (Figure 2) despite different environmental settings (e.g., lake and catchment sizes and bathymetry) influencing the sediment accumulation rates and patterns in the lakes (Tables S1–S5). Furthermore, the records seem to present regional rather than local SBC deposition trends as the trends at Kharby, Podvaty, and Vork 9 are consistent with the trend at PONE situated ca. 1000 km away. The deposition trend shows clear similarities to increasing Russian anthropogenic BC emission inventory trends^48^ (Figures S4a and S5c) from 1850 to 1990. As natural fire emissions are suggested to have remained quite constant throughout the study period^43^ (Figure S5), it seems that the observed SBC trend is largely driven by anthropogenic emissions in the source areas (Figure S3). In general, the observed SBC deposition trend and source composition seem driven by long-range (hundreds to thousands of km) transported BC onto which regional (tens to hundreds of km) signals are superimposed, particularly strongly between ca. 1930 and 1990, as discussed below.

#### 4.1.1 1800–1930

The Timan–Pechora Basin was mainly unpopulated before the discovery of coal, oil, and gas in the 1930s (and 1960s in the West-Siberian Basin), and thus, SBC deposition and its subtle increase were likely mostly influenced by long-range transport until the early 1900s. This is supported by the model results, showing that until the 1950s, the BC deposition at the study sites was dominated by European emissions with relatively little regional (oil and gas producing region) contribution (Figure 4f,g). The domestic sector produced the bulk of emissions in both Europe and former Soviet Union during this time period (Figure S4b,c).^48^

According to the radiocarbon analyses, ca. 25% of the deposited SBC originated from fossil fuel combustion between 1800 and 1950s (Figure 3). Peat was commonly used as a fuel source in the Soviet Union before the onset of coal, oil, and gas production. In 1928, over 40% of electric power in the Soviet Union was produced by peat.^49^ Peat is considered a fossil fuel due to its slow renewability of thousands of years, and in northern Eurasia, peats are on average ca. 5000–6000 years old.^50−52^ In radiocarbon measurements, the radiocarbon signal of SBC produced by combustion of 5000–6000 years old peat equals the signal of 50% fossil fuel-derived SBC. Hence, if closer to a half of SBC emissions in the study region resulted from peat combustion and the rest from wood combustion in the 1800s, then the result of 25% fossil fuel-derived SBC can be explained by peat combustion. Also, natural peatland fires releasing modern to thousands of years old carbon^53^ may have affected the radiocarbon composition throughout the record but are expected to have remained constant, similar to other wildfires.^43^ In addition, some fossil fuel-derived SBC may have been long-range-transported from Europe where industrialization had already started (Figure S4c).

#### 4.1.2 1930–1985

At the end of 1920s, rapid industrialization began in the Soviet Union. The economic development met problems (such as famine) and industrialization had to be first spurred by the available biomass-based energy. These events may potentially be seen as the dip in SBC fluxes around 1930 (Figure 2e) and a slight increase in the relative contribution of biomass sources to the SBC fluxes around 1930–1950 (Figure 3), although according to model results, the contribution of regional emissions to the observed BC deposition would still have been quite small during this time period (Figure 4g).

**Figure 5:**
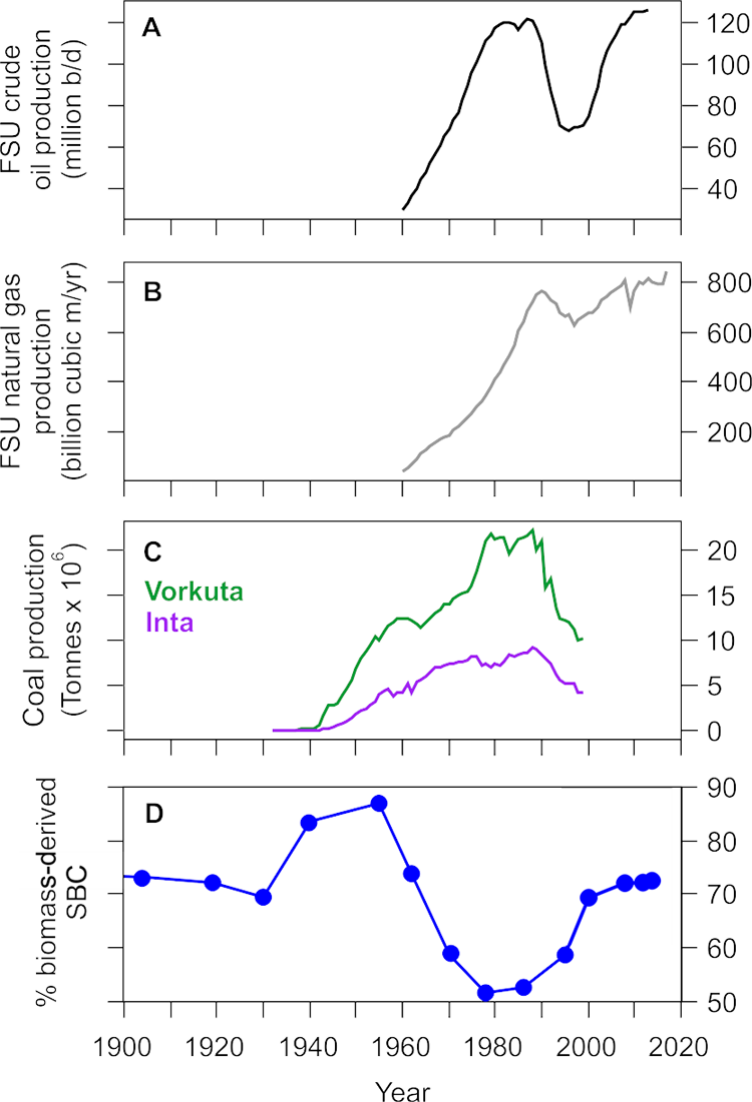
Former Soviet Union crude oil, natural gas, and coal production compared to the radiocarbon isotope data from Vork 9. (A) FSU crude oil production in millions of barrels per day,^62^ (B) FSU natural gas production in billion m^3^ year^–1^,^62^ (C) coal production in Vorkuta (green) and Inta (violet) in megatonnes,^63,64^ and (D) Vork 9 data on the percentage of biomass combustion-derived SBC, as in Figure 3b.

After the Second World War, industrialization expanded strongly in the Soviet Union and coal production increased rapidly in the Timan–Pechora Basin between 1945 and 1980s (Figure 5c). Also, oil and gas production increased rapidly after 1960, with oil production peaking from the late 1970s to late 1980s and gas production around 1990 (Figure 5a,b). The increase in fossil fuel production around the study lakes is clearly seen as an increase in SBC fluxes since 1960, peaking 1980–1990 in the sediment records (Figure 2). Simultaneously, the percentage of fossil fuel-originated SBC increases to 40–50% in the Vork 9 radiocarbon data (Figures 4 and 5d). The importance of the regional fossil fuel BC emissions to BC deposition in the study area is also evident in the modeling data, showing that the contribution of regional emissions to the BC deposited at Vork 9 increased from ca. 15 to around 50% from the 1950s to 1980s (Figure 4g).

#### 4.1.3 1986–2014

The fall of Soviet Union in 1991 had widespread adverse effects on the industrial production and the economy with coal production in the Timan–Pechora Basin falling dramatically (Figure 5c). Oil production started to substantially decrease already in the late 1980s while gas production was less affected (Figure 5a,b). The sediment SBC fluxes indicate a temporary sharp minimum around 1986 (Figure 2e), which was probably associated with the general economic decline at that time in the Soviet Union.^21^ However, the SBC fluxes quickly increased to similar or even higher levels than before the drop by the 1990s (Figure 2e). Thus, none of the sediment SBC records seem affected by the presumed rapid decline in the former Soviet Union anthropogenic BC emissions associated with the fall of the Soviet Union presented in emission inventories, and after ca. 1980, there is a clear mismatch between the modeled declines in BC deposition and emission inventory trends compared to the observed stable or increasing SBC flux trend in the study area (Figures 2, S4a, and S5c).

Gas production in the former Soviet Union started to increase again in 1997 and oil production around 2000 (Figure 5a,b), which may have contributed to the high and partly increasing observed SBC fluxes in the most recent sediment layers (Figure 2a,c). However, the radiocarbon data from Vork 9 indicate that the contribution of biomass sources to the deposited SBC has increased in the 1990s to 2000s compared to 1960–1990 (Figure 3). This likely reflects the sharp drop in coal production and consumption to pre-1950 levels (Figure 5c) and a potential switch to commonly used fuel wood in residential use in the study area.^45^ In addition, the results may imply an increased contribution of SBC deposition from wildfires in this time period, as further discussed in the Supporting Information S.13.

Until ca. 2000, the Vork 9 SBC deposition trend follows the other studied lakes, and thus, the Vork 9 source composition is also expected to represent all study lakes, but since ca. 2000, it is uncertain whether the Vork 9 source composition can be generalized to the study region.

### 4.2 Implications for Emission Inventories

Currently, there are significant uncertainties relating to BC sources in emission inventories which are directly propagated to emission inventory-based climate models, which in turn have challenges accurately assessing climatic impacts of BC.^3^ Generally, uncertainties in global BC emissions have been estimated to be a factor of 2.^54^

In bottom-up technology-based emission inventories,^48^ the Russian BC emissions are portrayed to have decreased sharply since the fall of the Soviet Union in 1991, while in updated emission inventories such as the presently used CMIP6, the decrease is more gradual and has turned to an increase and leveling off between 2000 and 2015 (Figures S4 and S5). The BC emission decrease has been used to explain the observed decrease in Arctic atmospheric BC concentrations since 1990.^4^ However, the current sediment data show that SBC deposition has stayed similar or has increased after 1991 in the study region. The study lakes are strongly influenced by emissions in the oil and gas producing regions, which present nearly half of total anthropogenic BC emissions from Russia according to the CMIP6 emission inventory (Figure S5c). In this region, BC emissions are suggested to have increased only slightly between 2000 and 2005 and declined since then (Figure S5c). However, SBC and elemental carbon deposition trends have increased after 1991 also in northern Finland lake sediments and a Svalbard ice core strongly influenced by much of the same Eurasian emissions as are the current study area (Figure S7).^7,8^ Together, these results imply either an offset between BC emission and observed SBC flux trends, for instance, due to meteorological process affecting BC scavenging efficiency variations^46^ or potential underestimations in BC emissions, emission factors, or the temporal trend in Russian emissions, particularly from the oil and gas producing area, since 1991 in emission inventories. The moderately increasing Russian, and particularly oil and gas producing region, CMIP6 emission trend (Figure S5c) seems clearly underestimated compared to the observed SBC flux trends between 2000 and 2015 (Figure 2).

Global CMIP6 emissions may potentially miss some emissions, particularly at a regional scale in the proximity to the study area. In the study area with nearby (within tens of kilometers) strong emission sources, it is likely that some emission sources may not be present in the model emission inventory or are smoothed out too much in the emission grid cell. Underestimations of modeled versus observed deposition have been related previously to errors in the quantity and spatial allocation of BC emissions in emission inventories driving the models.^10,45,55^ Unfortunately, the current data are not sufficient to pinpoint which of the emissions affecting the study sites may be underestimated in emission inventories between ca. 1990 and 2015, while the radiocarbon source data suggest that these are mainly of bio-fuel origin. Furthermore, more observational data are required to verify the SBC deposition trend and source composition in the area since 2000.

### 4.3 Significance of the Results in an Arctic Context

The here presented SBC fluxes significantly increase the available number and spatial coverage of Arctic BC deposition records (Figure 1a). Remarkably, unlike Arctic atmospheric monitoring data showing similar concentration trends across the Arctic since ca. 1990,^2,4^ the BC deposition records show considerable differences in temporal trends between Arctic sites. Greenland ice cores indicate peaking BC deposition around 1910 and a decrease to the present,^56^ and some Arctic European records show a similar 1910 peak as the Greenland records but also a distinct increase in BC deposition post-1970 (Figure S7),^7,8^ while the presented Russian records show a continued increase from ca. 1850 to the 2010s. The observed differences in BC deposition trends are likely caused by different source areas contributing to the records studied. The spatially varying BC deposition trends suggest that BC deposition patterns cannot necessarily be extrapolated over wider areas. Thus, to formulate efficient BC emission cuts and climate change mitigation strategies, more observations are urgently needed on BC deposition trends and specific emission types affecting these. Along with Asian emissions, Arctic shipping, and potentially flaring, emissions are the ones projected to increase in the Arctic and should therefore receive special attention.

Our results suggest that industrial (and associated residential, transportation, and energy) emissions may have had a notable and distinguishable influence on BC deposition in some parts of the Arctic during the 1900s, and Russian emission inventory data may need to be updated in BC emission trends since the 1990s, particularly for the Russian oil and gas producing region. The spatial influence of flaring emissions needs further assessment, as for instance, its effect on atmospheric BC concentrations in the Russian town of Tiksi have been shown to be small (ca. 6%) and overestimated in emission inventories,^10^ while flaring causes significant atmospheric BC peaks on Svalbard,^57^ but the current data do not show a significant contribution of fossil fuel sources to SBC deposition in the study region after 1990.

A possible explanation requiring further investigation for the lack of an evident flaring signal in the current sediments may be that the flaring-derived comparably small-sized hydrophobic BC particles are transported further away before deposition^58^ and are therefore not captured in the sediments. Accurate information on BC emissions and their trends from the Russian oil and gas producing region are essential as this area causes nearly half of the Russian anthropogenic BC emissions (Figure S5c). Our data suggests that also recently potentially increasing wildfire emissions from Russia may be underestimated in emission inventories. In Arctic climate change, BC emissions from Russia are particularly detrimental as they present a within-Arctic emission source causing 5 times stronger warming than the same amount of emissions at mid-latitudes.^59^

## Supporting Information Available

Site description and radiometric dating of lake sediment cores; detailed description on the SBC analytical methodology and an assessment of method accuracy and precision; total organic carbon and SBC results of the standard reference material; non-soot organic carbon (NS-OC) and SBC concentrations in the lake sediments and their correlations; SBC radiocarbon measurement results; evaluation of FLEXPART model uncertainties; modeled sensitivities of total BC deposition to surface emissions; emission inventory Russian and European anthropogenic BC emission trends and source sectors 1850–2000; CMIP6 BC emissions 1900–2015; comparison of observed and modeled sources for the SBC and BC deposited at the study sites; and Arctic BC deposition trends in previously published records (PDF)
