# Supplementary material for: Observed and Modeled Black Carbon Deposition and Sources in the Western Russian Arctic 1800–2014
Source: Environ Sci Technol. 2021 Mar 26;55(8):4368–77. doi: 10.1021/acs.est.0c07656 (PMC8154361; doi:10.1021/acs.est.0c07656)
Supplement: Supplementary file 1 — es0c07656_si_001.pdf [file es0c07656_si_001.pdf]

Supporting Information for

Observed and modelled black carbon deposition and sources in the western Russian Arctic 1800-2014

Meri M. Ruppel, [1,]** *Sabine Eckhardt,* [2] *Antto Pesonen,* [3,4] *Kenichiro Mizohata,* [5] *Markku J. Oinonen,* [4] *Andreas Stohl ,*[6] *August Andersson,* [7] *Vivienne Jones,* [8] *Sirkku Manninen,* [1] *Örjan Gustafsson* [7]

[1] Ecosystems and Environment Research Programme, Faculty of Biological and Environmental Sciences, University of Helsinki, FI-00014 Helsinki, Finland

[2] Norwegian Institute for Air Research (NILU), NO-2027 Kjeller, Norway

[3] Technology Center, Neste Corporation, FI-06101 Porvoo, Finland

[4] Laboratory of Chronology, Finnish Museum of Natural History – LUOMUS, University of Helsinki, FI-00014 Helsinki, Finland

[5] Division of Materials Physics, Department of Physics, University of Helsinki, FI-00014 Helsinki, Finland

[6] Department of Meteorology and Geophysics, University of Vienna, A-1090 Vienna, Austria

[7] Department of Environmental Science and the Bolin Centre for Climate Research, Stockholm University, SE-106 91 Stockholm, Sweden

[8] Environmental Change Research Centre, Department of Geography, University College London, London WC1E 6BT, United Kingdom

Corresponding author
*E-mail: meri.ruppel@helsinki.fi

Description of Supporting Information:

Number of pages: 24

Number of tables: 7

Number of figures: 7

Content included:

- Table of detailed description on study lakes
- Tables (4) of radiometric dating of lake sediments
- Details on analytical SBC quantification methodology
- Assessment of method accuracy and precision
- Table of total organic carbon and soot black carbon concentrations of various standard reference material
- Figure on non-soot organic carbon and soot black carbon concentration of the lake sediment samples and their correlation
- Table and figure of SBC radiocarbon source apportionment results
- Evaluation of FLEXPART model uncertainties
- Figure on modelled sensitivities of total BC deposition to surface emissions
- Figure on emission inventory Russian and European anthropogenic BC emissions and source sectors 1850–2000
- Figure on BC emissions 1900–1999
- Comparison of observed and modelled sources for the SBC and BC deposited at the study sites
- Figure on Arctic BC deposition trends in previously published records
- References

S. 1. Study area and lake sediment coring

The Timan-Pechora basin is dominated by lowland tundra with continuous permafrost while the West Siberian Basin constitutes a large wetland area reaching from Arctic tundra in the north to the southern edge of the taiga forest zone in the south. Table 1 presents general information on the study lakes. The Vork 9 and Kharby sediments were collected in 2014 and the Podvaty sediment core in 1998 from the Timan-Pechora Basin in the vicinity of the three major industrial centers Usinsk, Inta and Vorkuta (Fig. 1). The cores were collected with a Glew gravity corer.[1] The Kharby sediment was sampled at 0.5 cm resolution for the top 10 cm, and 1 cm resolution below 10 cm, while the Vork 9 sediment was cut at 0.5 cm resolution for the top 20 cm, and 1 cm resolution below. The Podvaty sediment was extruded at 0.5 cm intervals for the first 5 cm and then at 1cm intervals below. The lakes are located in a flat lowland tundra environment with continuous permafrost, and have no major river in- or outfluxes.

The PONE sediment was collected in 2006 from the Putorana Plateau, a more pristine environment than the Timan-Pechora Basin, although at ca. 200 km proximity to one of the most polluted mining cities of Russia, Norilsk, with a HON-Kajak corer,[2] and subsampled at 0.25 cm intervals. PONE is an unnamed lake that was given the code name PONE in a previous study[3]. Due to the lack of sediment material, PONE samples were combined so that BC quantifications could be made at 0.5 cm resolution. The lake is situated in mountainous open woodland in a hanging valley between two mountain ridges and has a single outflow stream.[3]

All sediment cores were collected at the deepest point of the lakes which was determined by transect water depth measurements with a hand-held echo sounder. At the deepest part of the lakes the potential sediment resuspension is expected to be lowest.

Table S1. General information on the studied lakes.

Lake name	**Kharby**	**Podvaty**	**PONE**	**Vork 9**
Location	67°31' N, 62°52' E	67°27' N, 63°05' E	68°08′ N, 92°12′ E	67°32' N, 62°45' E
Altitude (m a.s.l.)	122	116	596	140
Size (ha)	2000	44	0.9	1
Catchment size (ha)	500	na	16	na
Maximum depth (m)	15.5	8.7	5	8
Mean sedimentation rate (g cm^{-2} yr^{-1})	0.05	0.031	0.022	0.018
Coring year	2014	1998	2006	2014

S.2. Radiometric dating of lake sediments

The sediments were analyzed for ^{210}Pb, ^{226}Ra, ^{137}Cs, and ^{241}Am by direct gamma assay at the University College London Environmental Radiometric Facility. Details on the dating methodology are given in Refs [4–6].

None of the sediments were laminated or varved. However, the dating results of the study sediments showed no indication of bioturbation, hiatuses or vertical mixing of the sediments. Generally, bottom-dwelling species that would affect the dating of the sediments are quite rare in Arctic lake sediments.

Table S2. Artificial fallout radionuclide and ^{210}Pb concentrations, sedimentation rate and chronology of Kharby.

			Sedimentation rate			Chronology		
Depth	^{210}Pb	^{137}Cs				Date	Age	
cm	Bq Kg^{-1}	Bq Kg^{-1}	g cm^{-2} yr^{-1}	cm yr^{-1}	± %	AD	yr	±
0.25	375.82 ± 28.43	15.39 ± 3.42	0.087	0.783	8.8	2014	0	2
1.25	348.29 ± 21.04	21.04 ± 2.71	0.0898	0.703	7.2	2012	2	2
2.25	404.94 ± 26.95	27.27 ± 3.04	0.073	0.408	7.8	2011	3	2
3.25	360.7 ± 23.23	33.89 ± 3.01	0.0753	0.297	7.6	2008	6	2
4.25	397.33 ± 15.11	32.65 ± 1.97	0.0591	0.191	5.1	2003	11	2
5.25	324.05 ± 16.19	53.72 ± 2.51	0.0627	0.179	6.5	1998	16	2
6.25	283.5 ± 13.3	65.79 ± 2.39	0.0605	0.16	6.5	1992	22	2
7.25	221.06 ± 14.17	105.26 ± 3.15	0.0671	0.169	8.7	1986	28	2
8.25	186.21 ± 6.91	116.67 ± 1.71	0.0684	0.16	6.6	1980	34	2
9.25	158.55 ± 9.32	124.28 ± 2.46	0.0671	0.15	9.2	1973	41	2
10.5	166.45 ± 9.81	205.57 ± 3.16	0.0459	0.105	9.9	1963	51	2
11.5	111.83 ± 8.57	157.81 ± 2.72	0.059	0.139	13.4	1955	59	3
12.5	97.99 ± 9.17	77.7 ± 2.12	0.054	0.131	16.3	1948	66	3
13.5	83.85 ± 8.04	26.15 ± 1.36	0.0613	0.154	20	1941	73	4
14.5	95.18 ± 8.04	14.62 ± 1.11	0.0353	0.089	18.7	1932	82	5
15.5	73.02 ± 5	7.9 ± 0.6	0.0403	0.101	22.4	1921	93	6
17.5	49.22 ± 5.78	2.11 ± 0.56	0.0592	0.141	44.4	1905	109	9
19.5	45.86 ± 5.62	0	0.0403	0.086	55.4	1887	127	13
21.5	41.45 ± 4.93	0	0.0188	0.037	74.1	1853	161	24

Table S3. Artificial fallout radionuclide and ^{210}Pb concentrations, sedimentation rate and chronology of Podvaty.

			Sedimentation Rate			Chronology		
Depth	^{210}Pb	^{137}Cs				Date	Age	
cm	Bq Kg^{-1}	Bq Kg^{-1}	g cm^{-2} yr^{-1}	cm yr^{-1}	± %	AD	yr	±
0.25	226.6 ± 19.1	86.9 ± 6.3	0.051	0.34	10.9	1998	0	
2.25	238.8 ± 13.9	86.6 ± 2.9	0.037	0.20	8.0	1991	7	2
3.25	203.9 ± 8.1	98.6 ± 2.4	0.036	0.15	7,3	1986	12	2
4.25	150.9 ± 10.8	89.0 ± 2.9	0.037	0.12	10,8	1979	19	2
5.50	157.7 ± 10.8	99.6 ± 2.9	0.025	0.069	10.7	1963	35	2
6.50	110.5 ± 9.1	83.3 ± 2.3	0.024	0.060	14.6	1947	51	3
7.50	75.7 ± 3.8	43.6 ± 0.9	0.024	0.054	14.7	1930	68	5
8.50	53.2 ± 5.9	12.1 ± 0.9	0.022	0.048	14.7	1910	88	8
9.50	43.7 ± 4.8	6.8 ± 0.9	0.022	0.044	14.7	1889	109	13
10.50	35.3 ± 3.2	4.3 ± 0.8	0.022	0.045	18.2	1866	132	24

Table S4. Artificial fallout radionuclide and ^{210}Pb concentrations, sedimentation rate and chronology of PONE.

			Sedimentation rate			Chronology		
Depth	^{210}Pb	^{137}Cs				Date	Age	
cm	Bq Kg^{-1}	Bq Kg^{-1}	g cm^{-2} yr^{-1}	cm yr^{-1}	± %	AD	yr	±
0.5	221.63 ± 25.86	36.78 ± 3.83	0.0337	0.137	14.7	2002	4	2
1.75	240.35 ± 18.34	37.91 ± 2.81	0.0239	0.093	12.9	1993	13	2
2.75	150.57 ± 11.02	52.1 ± 1.92	0.0233	0.083	14.8	1981	25	3
3.25	95.65 ± 12.31	52.6 ± 2.52	0.0284	0.096	18.5	1976	30	4
3.75	121.95 ± 17.89	56.96 ± 3.31	0.0264	0.085	22.6	1970	36	5
4.75	86.37 ± 11.67	53.23 ± 2.15	0.0185	0.054	30.5	1954	52	8
6.25	39.13 ± 8.43	5.55 ± 0.98	0.0222	0.062	61.1	1927	79	17
7.75	35.8 ± 8.9	1.35 ± 0.93	0.01	0.026	88.2	1889	117	25

Table S5. Artificial fallout radionuclide and ^{210}Pb concentrations, sedimentation rate and chronology of Vork 9.

			Sedimentation rate			Chronology		
Depth	^{210}Pb	^{137}Cs				Date	Age	
cm	Bq Kg^{-1}	Bq Kg^{-1}	g cm^{-2} yr^{-1}	cm yr^{-1}	± %	AD	yr	±
0.25	242.5 ± 34.33	37.47 ± 6.39	0.0406	0.706	21.3	2014	0	2
1.25	394.89 ± 42.82	50.43 ± 8.27	0.0206	0.301	16.3	2012	2	2
2.25	276.57 ± 22.45	91.81 ± 5.96	0.0258	0.332	14.5	2008	6	2
3.25	346.53 ± 24.83	96.54 ± 5.8	0.0187	0.23	14.9	2005	9	2
4.25	325.66 ± 22.61	122.49 ± 5.95	0.0169	0.2	16.3	2000	14	2
5.25	316.16 ± 13.46	150.59 ± 3.81	0.0145	0.146	17.5	1995	19	3
6.25	302.87 ± 14.12	168.8 ± 4	0.0119	0.097	22.5	1986	28	3
7.25	269.78 ± 15.01	131.97 ± 4.25	0.0178	0.145	18.1	1981	33	3
8.25	232.92 ± 12.91	117.71 ± 3.49	0.0176	0.169	18.9	1976	38	3
9.25	170.77 ± 17.52	107.25 ± 5.3	0.0212	0.223	24.4	1972	42	3
10.25	153.17 ± 16.79	83.42 ± 3.74	0.0208	0.225	25.6	1968	46	3
11.25	151.75 ± 17.15	79.61 ± 3.67	0.0169	0.188	25.5	1964	50	4
12.25	134.9 ± 17.22	59.36 ± 3.22	0.0188	0.204	29.6	1960	54	4
13.25	109.75 ± 13.64	41.97 ± 2.36	0.02	0.212	29.9	1955	59	4
14.25	109.98 ± 12.26	18.64 ± 1.54	0.0173	0.155	29.2	1951	63	5
15.75	89.36 ± 8.43	12.84 ± 1.11	0.0157	0.126	30.4	1940	74	6
17.25	87.48 ± 10.67	3.36 ± 1.43	0.0107	0.086	38	1926	88	8
19.25	62.26 ± 11.36	0	0.0078	0.063	47.4	1899	115	13
21.5	48.19 ± 8.42	0	0.0033	0.026	60.2	1849	165	31

S.3. Soot Black Carbon (SBC) analysis with the chemothermal oxidation at 375 °C method (CTO-375)

The term black carbon comprises a myriad of carbonaceous particles formed under variable incomplete combustion conditions and from different fuel materials. These particles ranging from charred biomass to highly condensed and refractory soot owe different physical properties such as size, composition and structure. Thus, BC is not a precisely defined term. Currently, no analytical method is capable to quantify all BC particles simultaneously, and no standard method exists for BC quantification. Consequently, BC is an operational term and its precise definition depends on the method used for its quantification.[7,8]

Here, BC was quantified with the chemo-thermal oxidation method at 375 °C (CTO-375). The method effectively quantifies the most condensed high-refractory fraction of BC, the so called soot-BC (SBC).[9] SBC is formed by gas condensation in high-temperature flames of both biomass and fossil fuel burning when combustion temperatures are sufficient. Additionally, SBC may include some char-type BC that is a high-temperature combustion residue containing some morphological features of the burned material, such as Spheroidal Carbonaceous Particles (SCPs).[8] Generally, however, char-BC particles are less condensed forms of BC formed at lower combustion temperatures. As char-BC has lower thermal-oxidative stability it is mostly not quantified with the CTO-method.[9] Thus, SBC measurements represent a subset of total BC. Importantly, natural gas flaring (as well as for instance vehicle engines) produces BC only as a gas condensate in flames, and thus the CTO-375 method is well-applicable for our purposes to study the historical trend of flaring-derived BC, as well as all most refractory and smaller sized far travelling BC particles.

For the CTO-375 analyses and SBC quantification as in Refs[10-12] the dried sediments were homogenized to less than 100 µm particle size with a stainless steel ball grinder (Retsch, Mixer Mill 4000). To oxidize organic material, approximately 10 mg of sediment was precisely weighed into pre-combusted (12 h at 450 °C) silver capsules (5 × 8 mm), and combusted in a custom-made tube furnace at 375 °C for 18 hours under active airflow (200–300 mL min-1) at Stockholm University. Subsequently, carbonates were removed by in-situ microscale acidification with 1M HCl. Finally, the residual carbon, i.e. SBC, and nitrogen (N) concentrations were quantified with a Flash 2000 Organic Elemental Analyser (Thermo Fisher) at 950 °C at the University of Helsinki, Laboratory of Chronology. The measurements were calibrated, and the reliability and repeatability of the analysis determined, by including 5–6 standardized soil reference standard samples of known carbon and nitrogen content supplied by the device manufacturer in each analytical run of 32 samples.

To evaluate the potential risk of charring to the samples, the non-soot organic carbon (NS-OC) content of the samples was determined. Approximately 10 mg of each sediment sample was weighed into silver capsules and acidified with 1 M HCl, as above in the SBC analyses. The total organic carbon (TOC) content was quantified with the elemental analyzer and the NS-OC content of the samples determined by subtracting the amount of SBC from the TOC values. A clear positive correlation between NS-OC and SBC values could potentially indicate charring in the samples, as non-BC condensed organic matter could be charred and measured as SBC during the analysis. However, the CTO-375 method has been demonstrated to have a lower potential to give false positives compared to other BC quantification methods.[11,13] Also here, the SBC concentrations show no correlation with respective NS-OC concentrations, which strongly indicates that the SBC quantification was not affected by charring (Fig. S1).

S.4. Evaluation of CTO-375 method accuracy and precision

The accuracy of the CTO-375 method can be evaluated by comparing SBC results of selected standard reference material reported in previous studies using the same or similar methods. Wood char, melanoidin, Baltimore Harbor sediment (NIST SRM-1941b), urban dust (NIST SRM-1649b), and diesel particulate matter (NIST SRM-2975) samples were prepared along with the lake sediment samples. These reference materials were chosen as they include 1) BC-containing environmental matrices (Baltimore Harbor sediment, urban dust aerosol and diesel particulate matter), 2) laboratory-produced BC-rich materials (wood charcoal), and 3) potentially interfering materials originally devoid of BC but potentially creating BC during analysis (melanoidin). It is generally advised in the geochemical community to assess the BC analysis method accuracy and to specify the type of BC quantified by the analysis of these three reference material types in each study. This facilitates comparative analyses of BC in diverse environmental matrices and by different analytical methodologies. The wood char and melanoidin reference material were provided by Prof. Michael Schmidt, Univ. Zürich (https://www.geo.uzh.ch/en/units/2b/Services/BC-material.html) and the rest by the U.S. National Institute of Standards and Technology (NIST, Gaithersburg, MD, USA).

Our measurements and previously reported results of SBC and TOC concentrations of the five standard reference material samples are presented in Table S6. The SBC results of all standard materials are within the standard deviations of published mean data, and mostly have smaller relative standard deviations than previous studies. No SBC was detected in wood char that contains high amounts of organic carbon but no SBC, indicating that oxygen supply during the oxidation phase of the method was sufficient to avoid charring.

Table S6. Total organic carbon (TOC) and soot BC (SBC) values for five standard reference material tested in this study and from literature (TOC lit and SBC lit), and their standard deviations.

	**TOC** (%dw)	**SBC** (%dw)	**TOC lit** (%dw)	**SBC lit** (%dw)
Wood char	66.5 ± 0.25	n/a	69.9 ± 0.6[12] – 76 ± 0.18[9]	n/a
Marine Sediment (SRM-1941b)	3.17 ± 0.02	0.59 ± 0.02	2.97 ± 0.032[9] – 3.1 ± 0.1[24]	0.41 ± 0.08[24] – 0.60 ± 0.01[12]
Urban Dust (SRM-1649b)	18.49 ± 0.07	1.59 ± 0.02	16.8 ± 0.32[9] – 18.4 ± 0.4[12]	1.36 ± 0 07[11] – 1.83 ± 0.027[9]
Melanoidin	52.43 ± 0.73	3.22 ± 0.29	52.5 ± 0.14[12] – 61.9 ± 0.41[9]	2.5 ± 0.22[9] – 3.82 ± 0.17[12]
Diesel PM (SRM-2975)	86.79 ± 0.32	68.41 ± 0.72	84.4 ± 0.9[9] – 87.2 ± 0.3[11]	63 ± 4.1[25] – 68.9 ± 0.79[12]

%dw: percent of dry mass, n/a: below detection limit. Most literature values for Urban Dust are of SRM 1649a.

Samples of the sediment reference material (SRM-1941b) were included in each lake sediment CTO-375 and elemental analyzer run to check for possible errors during the chemothermal oxidation or in the elemental analysis. The method precision was ascertained by analyzing randomly selected sediment samples in duplicates and triplicates. The average relative standard deviation was 3.7 % (range 0.06–9.5 %) for 15 different duplicate and triplicate SBC samples prepared from the lake sediments. Additionally, the relative standard deviation of SBC in the Baltimore Harbor sediment (SRM-1941b) was 3.9 % in 22 samples. Thus, the method precision can be estimated to be around 3.8 %. The method precision here is thereby better than previously reported values of up to 10 % [14] and 23 % [15] for SBC samples, but slightly lower than previously using the same instrumentation (1.7 %)[12]. Where duplicate to triplicate results are available for the SBC results, the mean concentrations are reported and error bars shown in Fig. S1. Several blank samples were prepared to check for contamination or cross-contamination of the samples during the sample preparation and elemental analysis. All had carbon contents below the detection limit (0.01 %, given by the device manufacturer).

S.5. SBC and NS-OC concentrations and their correlations

Figure S1 presents the SBC and NS-OC concentrations (g gDw^{-1}) of the lake sediment samples. SBC represents a minor component of sediment and its concentrations may be strongly diluted or concentrated by the varying influx of other sediment components such as organic and mineral material. Consequently, SBC concentrations may vary substantially with time and from one lake to another, and their significance as such is minor for this study. However, the charring potential of the samples was assessed by testing the correlation between the SBC and NSOC contents of the samples. Based on the correlations none of the sediment cores suggest potential charring.

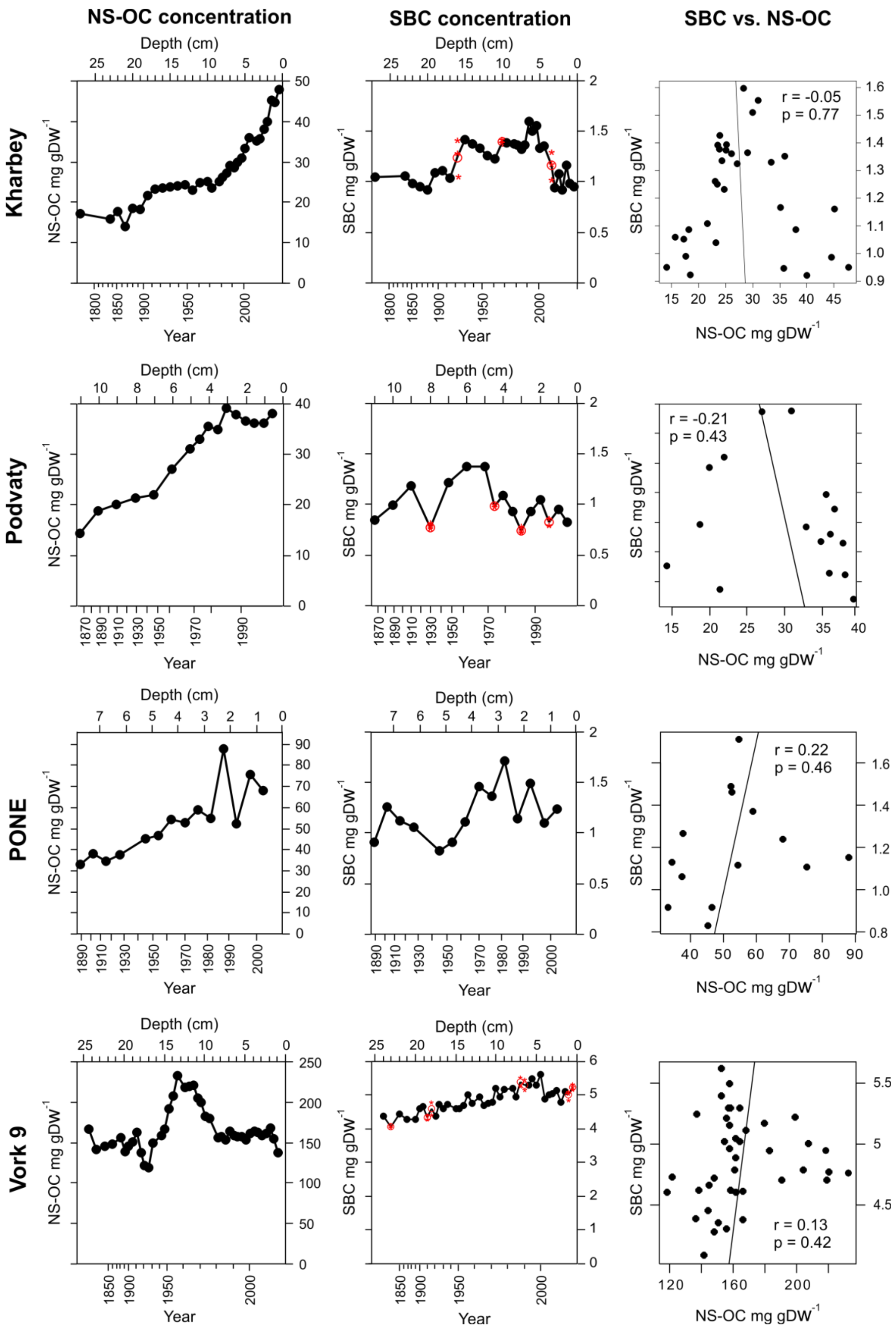

Figure S1. Non-soot organic carbon (NS-OC) and soot black carbon (SBC) concentrations (mg gDw^{-1}) and their correlation. Note that the axes are not the same for all lakes. Samples of duplicate and triplicate analyses are shown as red asterisks and their average as an open red circle.

S.6. Detailed description on the gas-ion-source instrumentation in the radiocarbon isotope analysis at the Accelerator Laboratory of the University of Helsinki

The gas injection system of the Helsinki Accelerator Mass Spectrometry hybrid ion source was built to handle gas samples from 12 storage containers. It uses a pneumatically controlled syringe to inject the CO_2, and helium as carrier gas to carry the CO_2, through the capillary pipes. The CO_2 samples are moved from the sample intake, and between storage containers and the syringe, by cryogenic traps with liquid nitrogen. The efficiency of sample transportation done with this freezing - warming process is almost 100 %. Gas pre-pressures for He and CO_2 were set to 3 bar. With these pressures the carbon current from the ion source is 12 µA. Titanium cathodes were used and they were pre-sputtered for five minutes to remove carbon contaminants from the cathode surface. A similar gas-ion-source system for radiocarbon measurements has been presented in previous work.[16]

Subsequently, the 16 sediment samples selected from the Vork 9 core were measured for their radiocarbon content with Accelerator Mass Spectrometry (AMS).

S.7. ^{14}C analyses with traditional AMS methodology

In addition to the 16 Vork 9 samples analyzed with the gas-ion-source, 6 parallel samples from the same sediment core were prepared by traditional graphitization for the ^{14}C analyses. For this, nine sediment subsamples of ca. 11 mg size were prepared with the CTO-375 method and pooled together into one CO_2 trap, and subsequently graphitized by the Helsinki Adaptive Sample preparation line,[17] and the radiocarbon content determined with AMS.

This was done to compare the results of the newly deployed hybrid ion source instrumentation with the more traditional ^{14}C analyses including the graphitization step (for results, see S.9.). The gas-ion-source step in the AMS analyses has several advantages compared to the traditional measurements using graphitization as a sample preparation step, as a) the required sample size for the gas-ion-source measurements is roughly one tenth of the traditional method and thereby extremely valuable for e.g. Arctic samples devoid of high BC concentrations, and b) because one sample preparation step potentially causing artificial isotopic fractionation (graphitization) is eliminated with the gas-ion-source in comparison to the old methodology. Samples are also easier and faster to prepare and the AMS measurements are substantially faster with the gas-ion-source compared to the traditional methodology.

S.8. Calculation of the bio-based carbon percentage of the extracted SBC, i.e. radiocarbon source apportionment

The radiocarbon source apportionment of the 16 selected Vork 9 sediment core samples was based on first determining the radiocarbon content (given as pMC, i.e. percentage modern carbon[18]) of the samples by AMS and then calculating the percentage of the bio-based carbon (bio % SBC) (Table S7). The bio-based carbon percentage calculations of the extracted SBC were performed as described in the standard procedure ASTM D6866-20[19] by dividing the quantified pMC by an atmospheric reference pMC value and multiplying the result by 100.

$$Bio\ \%\ SBC = 100 \times \frac{pMC_{sample}}{pMC_{atm\ REF}}$$

The atmospheric radiocarbon concentration of the year 1950 is the reference year, to which all radiocarbon measurements are compared. This content is defined as 100 pMC (percent modern carbon, or 100 % of the radiocarbon content compared to 1950's level). Fossil material on the other hand has a value of 0 pMC, as it is completely devoid of radiocarbon. However, nuclear testing starting in the 1950s increased globally the naturally occurring atmospheric radiocarbon content which peaked in the 1960s and has subsequently decreased to 1950s values again. All biomass growing during the nuclear bomb testing period incorporated increased radiocarbon levels, depending on their growing period. Here, the naturally varying atmospheric level of radiocarbon was compensated by applying an atmospheric correction factor. As the biomass component of the samples is expected to mainly consist of wood, and the turnover time of similar northern environment biomass has been estimated to be approximately 20 years in previous studies,[20,21] a model for average biomass radiocarbon content was created. For the model, the running averages of known atmospheric radiocarbon level of the previous 20 years were calculated based on the data of IntCal13[22] and Ref[23], and the radiocarbon level corresponding to the year which each specific sediment sample approximately represented, was applied as an atmospheric correction factor. Thus, the bio % SBC results were calculated as the biomass-derived carbon mass percentages of the total carbon of the SBC samples. Potential error sources affecting the calculations are those inherent to the pMC measurements, the assumption of the turnover time of biomass (ca. 20 years), and sediment dating uncertainties.

A major advantage of the radiocarbon-based source attribution is the analysis on the deposited BC particle itself, while previous source attribution has used, for instance, chemical speciation of associated major ions and metal particles in snow[23–26] that may behave differently than BC during long-range transportation to the Arctic.[27]

S.9. Radiocarbon measurement results of selected Vork 9 sediment SBC samples

Table S7 presents measured pMC values and calculated biomass-derived percentages of the extracted SBC in the lake sediment samples of Vork 9. 16 samples were measured with the gas-ion-source AMS system and 6 with the traditional preparation procedure including graphitization. Also included are 2 measurements of the SBC extracted from NIST SRM-1941b.

Table S7. Running average of atmospheric reference pMC value of the previous 20 years of sampling date, measured pMC values, and calculated biomass-derived percentage of extracted SBC (bio % SBC) of the selected Vork 9 sediment samples.

Sample depth (cm)	Date	Atm ref pMC value	pMC value	pMC error	bio % SBC	bio SBC error %
0.0-0.5	2014	107.59	77.6	2.1	72.1	2.0
1.0-1.5	2012	108.63	78.0	2.0	71.8	1.9
2.0-2.5	2008	110.86	79.6	1.5	71.8	1.4
4.0-4.5	2000	116.89	81.0	1.6	69.3	1.4
5.0-5.5	1995	122.63	71.9	1.0	58.6	0.9
6.0-6.5	1986	139.61	73.1	1.6	52.4	1.2
7.5-8.0	1978	147.09	75.7	1.6	51.5	1.1
9.5-10.0	1970	130.35	76.4	1.7	58.6	1.4
11.5-12.0	1962	103.62	76.6	1.3	73.9	1.3
13-13.5	1955	97.80	84.9	1.5	86.8	1.5
15.5-16	1940	98.20	82.2	2.2	83.7	2.3
16.5-17	1930	98.50	68.4	1.4	69.4	1.5
17.5-18	1919	98.86	71.4	1.0	72.2	1.1
18.5-19	1905	98.95	72.4	1.3	73.2	1.3
21-22	1849	98.62	73.8	1.5	74.8	1.6
23-24	1815	98.08	73.5	1.1	75	1.1
1.0-1.5 GRAPHITIZED	2012	108.63	75.7	0.4	69.1	0.3
5.0-5.5 GRAPHITIZED	1995	122.63	81.0	0.6	66.3	0.3
9.5-10.0 GRAPHITIZED	1970	130.35	76.1	0.6	58.6	0.3
13-13.5 GRAPHITIZED	1955	97.80	75.1	0.6	76.1	0.4
17.5-18 GRAPHITIZED	1919	98.86	75.3	0.6	75.5	0.4
23-24 GRAPHITIZED	1815	98.08	74.5	0.4	75.4	0.3
SRM-1941 b 1	NA		13.5	0.5	11.0	0.4
SRM-1941 b 2	NA		14.0	0.8	11.4	0.7

SBC source apportionment results obtained with these methods generally agree well, and the higher resolution gas-ion-source samples clearly support and expand on the pMC trend also observed in the fewer graphitized samples (Fig. 4 and Table S7).

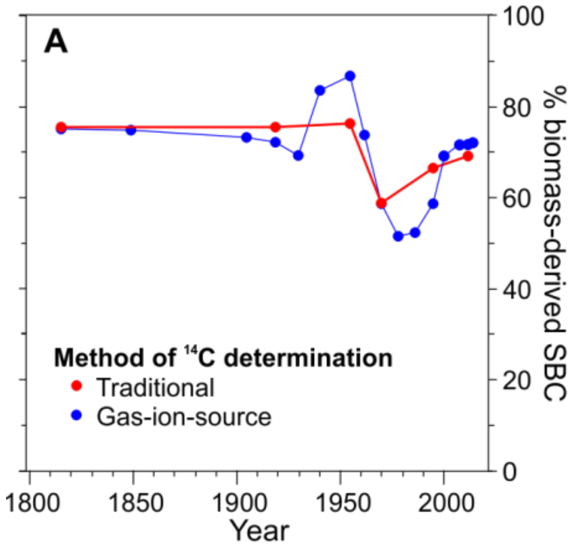

Figure S2. The biomass-derived SBC content of the Vork 9 sediment samples prepared for the AMS analysis by the gas-ion source step (*blue*), and the traditional procedure including graphitization (*red*).

S.10. Evaluation of FLEXPART model uncertainties

The Lagrangian particle dispersion model FLEXPART is widely used around the world,[28] and has been shown to capture well both BC transport to the Arctic,[29–32] as well as Arctic atmospheric BC concentrations and their seasonality.[33–35]

When comparing BC observations with FLEXPART model outputs it seems that discrepancies can be reduced significantly by changing the emission input data.[32,34] In particular, in northern Asia, uncertainties in the available emission inventories are large, and the available bottom-up emission inventories differ substantially both in magnitude and spatial disaggregation.[32] Thus, a substantial part of the uncertainties of the FLEXPART model outputs relate to the used emissions and their uncertainties.

The model uncertainties, other than the emission inventory uncertainties, consist mainly of two uncertainties: 1) uncertainties related to the meteorological input data (e.g., errors in winds, precipitation, etc.) and 2) errors in transport and removal within FLEXPART.

Errors of the meteorological input data could be assessed, at least to some extent, by using different meteorological input data sets. This could be done, for instance, by running an ensemble of model simulations based on different meteorological input data. However, to our knowledge no ensemble of meteorological analyses is available for the long time period (1900-1999) covered by our study. Meteorological ensembles are normally only available for operational analyses (the most recent but shorter ERA5 reanalysis also comes with a mini-ensemble), and even then it is not clear whether these ensembles can quantitatively describe the uncertainty in the transport calculations. It is beyond the

scope of the current study to perform ensemble model simulations to test the influence of the meteorological input data. However, it is likely that these uncertainties have a larger influence on the BC deposition variability on synoptic time scales than on deposition rates averaged over longer time periods since systematic biases in the meteorological model (e.g., of precipitation rates) are not contained in the ensemble uncertainty.

Errors in transport and removal within FLEXPART can be assessed by varying critical parameters in FLEXPART, especially related to dry and wet deposition. For instance, a relative model–observation mismatch of 32 %–43% for a three year study period at high latitudes, created by perturbation of scavenging coefficients for BC in the simulated concentrations, has been shown.[32] As far as we know this is the only study that has evaluated uncertainties of the model itself, in particular scavenging efficiencies. Such studies are valuable to assess uncertainties of FLEXPART. However, these uncertainty estimates were made only for atmospheric BC concentrations[32], and may not be directly applicable to BC deposition.

Model validation exercises require comparison with observational data. Unfortunately, BC deposition observations are currently scarce in the Arctic. To our knowledge, only one study[36] has quantified BC deposition from falling snow in the Arctic (Svalbard) for one winter season. For instance, ice core records or the current SBC flux data are not optimal for exact model validation as ice core and sediment BC deposition fluxes are affected by post-depositional processes and are not representative of only atmospherically deposited BC (although ice cores much better than sediments) and may have, for instance, dating problems. Thus, also the evaluation of uncertainties inherent for the FLEXPART model is beyond the scope of this study.

Importantly, the above listed uncertainties of the FLEXPART model and the input data mostly affect the exact amount of simulated BC deposition. Thus, as mentioned in the main text, we do not compare the exact values of modelled BC deposition and observed SBC fluxes. The listed uncertainties most likely do not affect the modelled BC deposition *trend*, and especially not in the yearly to decadal timescale which is relevant for our study. Thus, the potential model uncertainties should not affect the outcomes or discussion of the paper.

S.11. Sensitivities of total (wet and dry) BC deposition to surface emissions modelled with FLEXPART

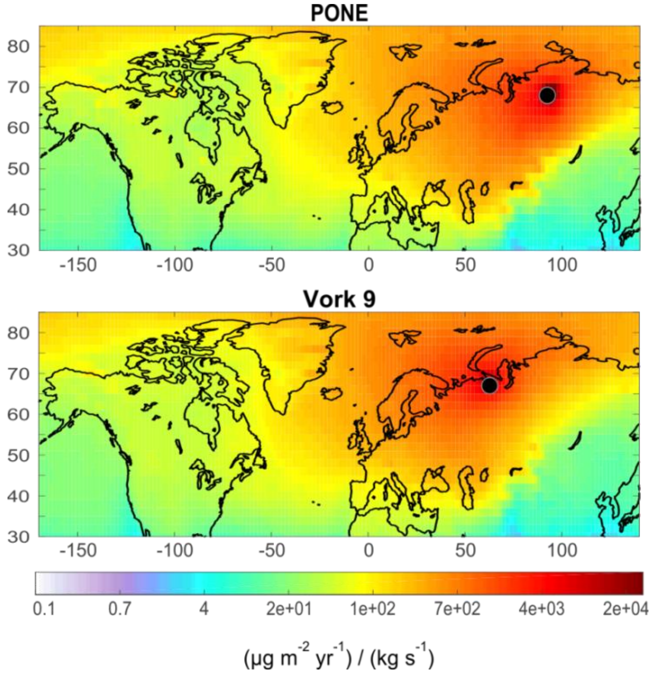

Figure S3. Footprint sensitivity of total (wet and dry) deposition to surface emissions [(μg m^{-2} yr^{-1})/(kg s^{-1})] for a BC tracer averaged over 100 years for the study locations PONE (upper panel) and Vork 9 (lower panel), marked with black dots. Notice the logarithmic scale.

S.12. Anthropogenic BC emission inventory data

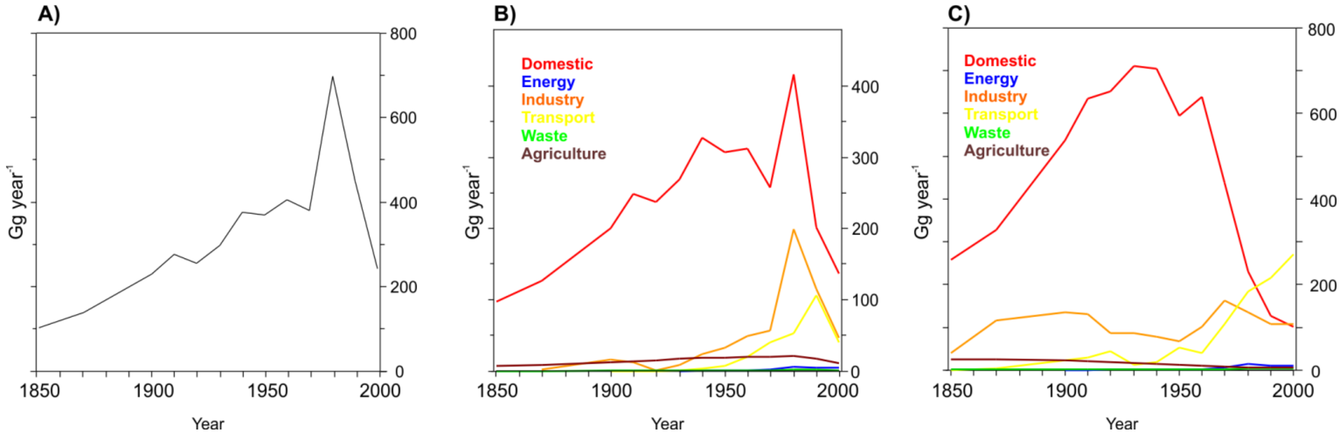

Figure S4. Russian (including Ukraine and Belarussia) and European anthropogenic BC emission data (in Gg $year^{-1}$) from 1850 to 2000.[38] A) Total BC emissions from Russia, Ukraine and Belarussia. B) BC emissions by sector (domestic, energy production, industry, transport, waste burning, and agricultural waste burning) from Russia, Ukraine and Belarussia. C) BC emissions by sector from Europe.

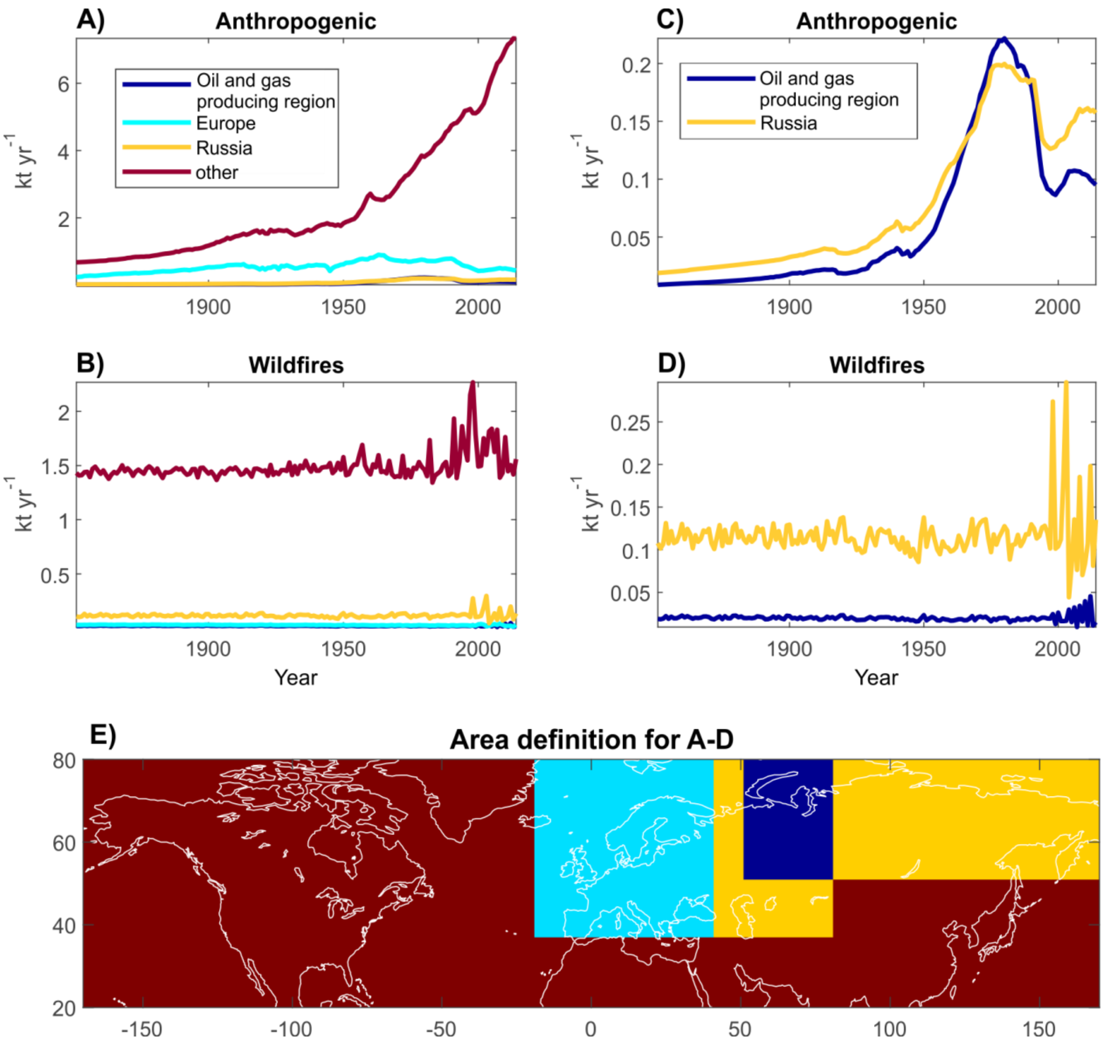

Figure S5. Anthropogenic[42] and wildfire[39] BC emissions (kt yr^{-1}) from 1850 to 2015 by defined areas used in this study. Emission data from CMIP6. Note, that since 1997 the wildfire emissions are derived from satellite data instead of fire proxies (such as charcoal records) and thus the yearly variations are higher than for 1850-1996 (for more details see the original study[39]). "Other" means the rest of the world.

S.13. Comparison of observed and modelled sources for the SBC and BC deposited at the study sites

The emissions used for the FLEXPART modelling included wildfire and anthropogenic BC emissions as shown above in Fig. S5. Optimally, these two emission groups of the emission inventory would be compared to the observed SBC radiocarbon-based biomass and fossil fuel-derived source classes. Unfortunately, such comparison is not feasible as they do not represent same emissions. For instance,

the radiocarbon-based biomass sources contain both emissions from wildfires and fuel woods, while fuel woods are classified as anthropogenic emissions in the emission inventory. Furthermore, peat combustion produces, depending on the age of the peat, a varying mixture of biomass and fossil fuel-derived emissions that are separated by the radiocarbon measurements, but are grouped as wildfire emissions in total in the used CMIP6 emission inventory. The anthropogenic emission sources are not further divided into sub-categories (such as residential, industry, transportation etc.) for 1900 to 1999 in the CMIP6 emissions. Thus, it is impossible to directly compare the source attributions made by the model and produced by the observations.

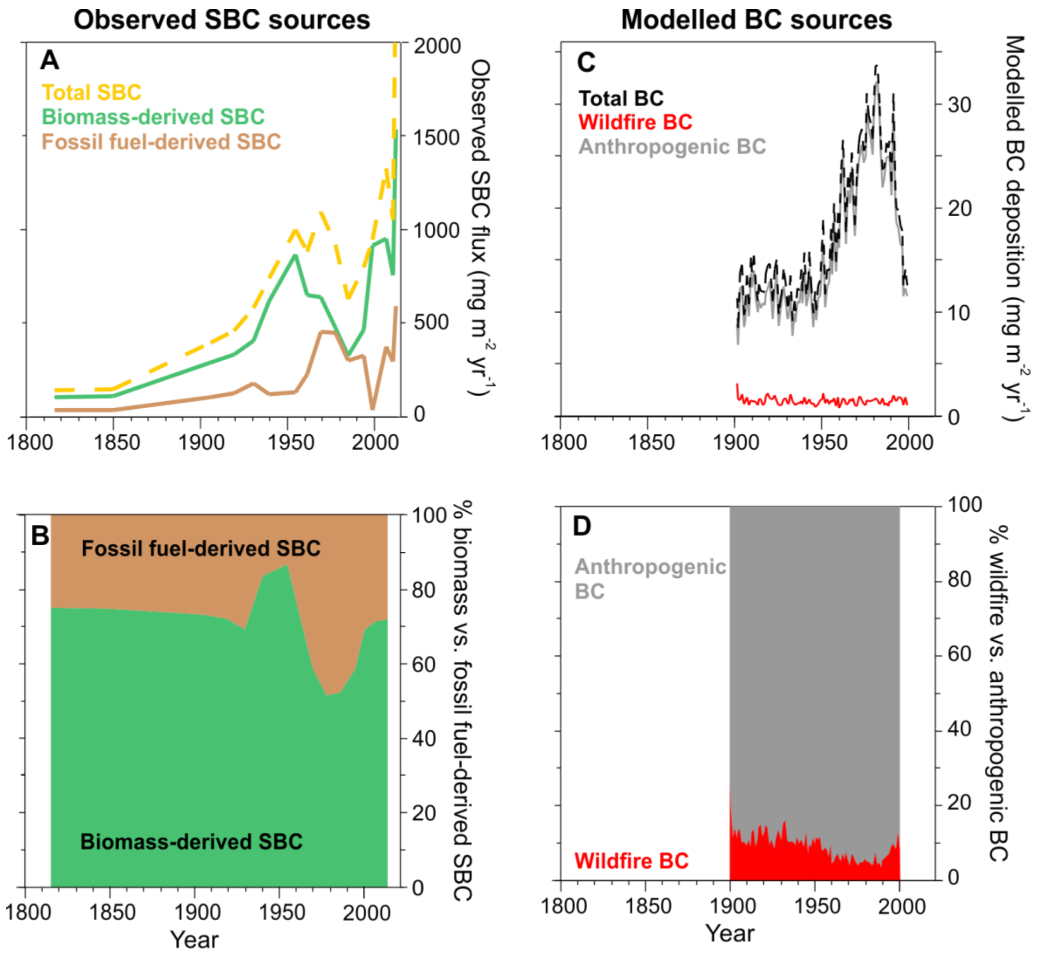

Figure S6. Observed SBC flux radiocarbon sources compared to modelled BC deposition sources to Vork 9. A) The temporal trend of SBC fluxes derived from biomass and fossil fuel sources. B) The contribution (%) of biomass vs. fossil fuel-derived sources to total SBC based on radiocarbon measurements of SBC. C) The temporal trend of modelled wildfire vs. anthropogenic sources to the BC deposited to Vork 9. D) The contribution (%) of wildfire vs. anthropogenic sources to total BC deposited at Vork 9.

Figure S6 shows that according to the radiocarbon measurements ca. 70 % of the SBC fluxes in the Vork 9 sediment originated from biomass combustion between ca. 1850 and 2014, while the modeling results suggest that between 1900 and 1999 only ca. 10 % of BC deposited at the site originated from wildfires. As mentioned, these source categories contain partly different sources.

Comparison of the observed and modelled results may suggest that the biomass-derived SBC contains to a majority SBC produced by combustion of fuel wood and other modern biomass. Indeed, BC emission from fuel wood combustion contributed around 61 % of the total residential BC emissions.[37] Thus, between 1850 to 2000 fuel wood combustion may constitute the bulk of biomass-derived SBC in our sediment record, as the domestic (i.e. residential) sector has caused by far the highest anthropogenic BC emissions from Russia,[38] particularly between 1850 to 1970 (ca. 80 %, Fig. S4b). Consequently, it seems that wildfires may have had only a minor contribution to SBC fluxes and their temporal trend in the Vork 9 sediment record. However, we do not have any information on the temporal development of fuel wood use in Russia from 1850 to 2000 and thereby we cannot verify this hypothesis. As the currently best available wildfire database[39] does not suggest an increase in Russian wildfire BC emissions during the last few decades, we assume that the observed increase of biomass-derived SBC fluxes in the sediment record between ca. 1980 and 2014 (Fig. S6) is mainly caused by a sharp drop in coal production and consumption and a potential switch to fuel wood in residential use in the study area in this time period. However, we also cannot preclude a possible increase in wildfire emissions in recent decades missed by emission inventories. Thus, our radiocarbon source apportionment data may suggest that BC emissions from wildfires are potentially underestimated in emission inventories, particularly since 1990. After 1990 our radiocarbon data show increased contribution of biomass-derived BC to SBC, but at the same time residential combustion has lost its dominance as the anthropogenic BC emitter (Fig. S4b) and thus the observed biomass-derived SBC is not likely only of residential origin.

S.13. Previously published data on BC deposition in the European Arctic

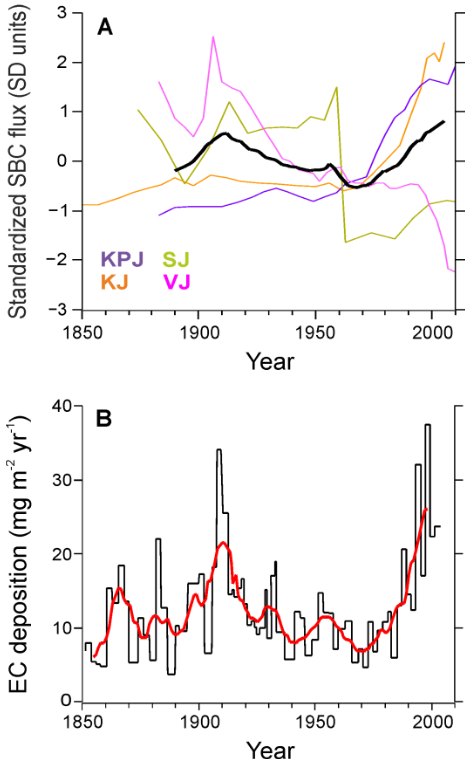

Figure S7. Standardized SBC fluxes in four Northern Finland lake sediments compared to elemental carbon (EC) deposition in a Svalbard ice core.[12]

REFERENCES

1. Glew, J. R. A new trigger mechanism for sediment samplers. *J. Paleolimnol*. **1989**, *2*, 241–243.

2. Renberg, I. The HON-Kajak sediment corer. *J. Paleolimnol.*, **1991**, *6*, 167–170.

3. Self, A. E.; Jones, V. J.; Brooks, S. J. Late Holocene environmental change in arctic western Siberia. *Holocene* **2015**, *25*, 150–165, DOI: 10.1177/0959683614556387.

4. Appleby, P. G.; Oldfield, F. The calculation of 210Pb dates assuming a constant rate of supply of unsupported 210Pb to the sediment. *Catena* **1978**, *5*, 1–8.

5. Appleby, P. G.; Nolan, P. J.; Gifford, D. W.; Godfrey, M. J.; Oldfield, F.; Anderson, N. J.; Battarbee, R. W. 210Pb dating by low background gamma counting. *Hydrobiologia* **1986**, *141*, 21–27.

6. Appleby, P. G.; Richardson, N.; Nolan, P. J. Self-absorption corrections for well-type germanium detectors. *Nucl. Inst. & Methods B* **1992**, *71*, 228–233.

7. Petzold, A., Ogren, J. A., Fiebig, M., Laj, P., Li, S.-M., Baltensperger, U., Holzer-Popp, T., Kinne, S., Pappalardo, G., Sugimoto, N., Wehrli, C., Wiedensohler, A., Zhang, X.-Y.: Recommendations for reporting "black carbon" measurements, *Atmos. Chem. Phys*. **2013**, *13*, 8365–8379, https://doi.org/10.5194/acp-13-8365-2013.

8. Rose, N. L.; Ruppel, M. Environmental archives of contaminant particles. In: Blais JM, Rosen MR, Smol JP (eds.), Environmental Contaminants. **2015**, Developments in Paleoenvironmental Research 18, Springer, Dordrecht, pp 187–221, doi: 10.1007/978-94-017-9541-8_9.

9. Elmquist, M.; Cornelissen, G.; Kukulska, Z.; Gustafsson, Ö. Distinct oxidative stabilities of char versus soot black carbon: Implications for quantification and environmental recalcitrance. *Glob. Biogeochem. Cycles* **2006**, *20* (2); DOI 10.1029/2005GB002629.

10. Gustafsson, Ö.; Haghseta, F.; Chan, C.; Macfarlane, J.; Gschwend, P. M. Quantification of the dilute sedimentary soot phase: Implications for PAH speciation and bioavailability. *Environ. Sci. Technol.*, **1997**, *31* (1), 203–209.

11. Gustafsson, Ö.; Bucheli, T. D.; Kukulska, Z.; Andersson, M.; Largeau, C.; Rounzaud, J.-N.; Reddy, C. M.; Eglinton, T. I. Evaluation of a protocol for the quantification of black carbon in sediments. *Glob. Biogeochem. Cycles*, **2001**, *15* (4), 881–890.

12. Ruppel, M. M., Gustafsson, Ö., Rose, N. L., Pesonen, A., Yang, H., Weckström, J., Palonen, V., Oinonen, M. and Korhola, A.: Spatial and temporal patterns in black carbon deposition to dated Fennoscandian Arctic lake sediments from 1830 to 2010, *Env. Sci. Technol*., **2015,** *49*, 13954−13963, doi: 10.1021/acs.est.5b01779.

13. Hammes, K.; Schmidt, M. W. I.; Smernik, R. J.; Currie, L. A.; Ball, W. P.; Nguyen, T. H.; Louchouarn, P.; Houel, S.; Gustafsson, Ö.; Elmquist, M.; Cornelissen, G.; Skjemstad, J. O.; Masiello, C. A.; Song, J.; Peng, P.; Mitra, S.; Dunn, J. C.; Hatcher, P. G.; Hockaday, W. C.; Smith, D. M.; Hartkopf-Fröder, C.; Böhmer, A.; Lüer, B.; Huebert, B. J.; Amelung, W.; Brodowski, S.; Huang, L.; Zhang, W.; Gschwend, P. M.; Flores-Cervantes, D. X.; Largeau, C.; Rouzaud, J.-N.; Rumpel, C.; Guggenberger, G.; Kaiser, K.; Rodionov, A.; Gonzalez-Vila, F. J.; Gonzalez-Perez, J. A.; de la Rosa, J. M.; Manning, D. A. C.; López-Capél, E.; Ding, L.. Comparison of quantification methods to measure fire-derived (black/elemental carbon in soils and sediments using reference materials from soil, water, sediment and the atmosphere. *Glob. Biogeochem. Cycles* **2007**, *21*, GB3016.

14. Muri, G.; Wakeham, S. G.; Rose, N. L. Records of atmospheric delivery of pyrolysis derived pollutants in recent mountain lake sediments of the Julian Alps (NW Slovenia). *Environ. Poll.* **2006**, *139* (3), 461–468.11.

15. Bogdal, C.; Bucheli, T. D.; Agarwal, T.; Anselmetti, F. S.; Blum, F.; Hungerbühler, K.; Kohler, M.; Schmid, P.; Scheringer, M.; Sobek, A. Contrasting temporal trends and relationships of total organic carbon, black carbon, and polycyclic aromatic hydrocarbons in rural low-altitude and remote high-altitude lakes. *J. Environ. Monit.* **2011**, *13*, 1316–1326; DOI 10.1039/c0em00655f.

16. Xu, Sheng; Dougans, Andrew; Freeman, Stewart P. H. T.; Maden, Colin; Loger, Roger, Nuclear Instruments and Methods in Physics Research Section B, **2007**, *259* (1), 76−82, doi.org/10.1016/j.nimb.2007.01.311.

17. Palonen, V. Pesonen, A., Herranen, T., Tikkanen, P., Oinonen. M. HASE – The Helsinki adaptive sample preparation line. Nuclear Instruments and Methods in Physics Research Section B: Beam Interactions with Materials and Atoms **2013**, *294*, 182–184, doi.org/10.1016/j.nimb.2012.08.056.

18. Stuiver, M., Polach, H. A. Discussion: Reporting of 14C Data. Radiocarbon, **1977**, *19* (3), 355−363.

19. ASTM 2020. ASTM D6866-20, Standard Test Methods for Determining the Biobased Content of Solid, Liquid, and Gaseous Samples Using Radiocarbon Analysis, ASTM International, West Conshohocken, PA, **2020**, www.astm.org

20. Mouteva, G. O.; Czimczik, C. I.; Fahrni, S. M.; Wiggins, E. B.; Rogers, B. M.; Veraverbeke, S.; Xu, X.; Santos, G. M.; Henderson, J.; Miller, C. E.; Randerson, J. T. Black carbon aerosol dynamics and isotopic composition in Alaska linked with boreal fire emissions and depth of burn in organic soils. *Global Biogeochem. Cycles* **2015**, *29*, 1977–2000, doi:10.1002/2015GB005247.

21. Zencak, Z.; Elmquist, M.; Gustafsson, Ö. Quantification and radiocarbon source apportionment of black carbon in atmospheric aerosols using the CTO-375 method. *Atmos. Environ.* **2007**, *41*, 7895–7906.

22. Reimer, P., Bard, E., Bayliss, A., Beck, J., Blackwell, P., Ramsey, C., Buck, C. E., Cheng, H., Edwards, R. L., Friedrich, M., Grootes, P. M., Guilderson, T. P., Haflidason, H., Hajdas, I., Hatté, C., Heaton, T. J., Hoffmann, D. L., Hogg, A. G., Hughen, K. A., Kaiser, K. F., Kromer, B., Manning, S. W., Niu, M., Reimer, R. W., Richards, D. A., Scott, E. M., Southon, J. R., Staff, R. A., Turney, C. S. M., Van der Plicht, J. IntCal13 and Marine13 Radiocarbon Age Calibration Curves 0–50,000 Years cal BP. *Radiocarbon* **2013**, *55*, 1869−1887, doi:10.2458/azu_js_rc.55.16947.

23. Hua, Q., Barbetti, M., Rakowski, A. Atmospheric Radiocarbon for the Period 1950–2010. *Radiocarbon* **2013**, *55*, 2059-2072. doi:10.2458/azu_js_rc.v55i2.16177

24. McConnell, J. R.; Edwards, R.; Kok, G. L.; Flanner, M. G.; Zender, C. S.; Saltzman, E. S.; Banta, J. R.; Pasteris, D. R.; Carter, M. M.; Kahl, J. D. W. 20th century industrial black carbon emissions altered arctic climate forcing. *Science* **2007**, *317*, 1381–1384; DOI 10.1126/science.1144856.

25. Hegg, D. A.; Warren, S. G.; Grenfell, T. C.; Doherty, S. J.; Clarke, A. D. Sources of light-absorbing aerosol in arctic snow and their seasonal variation. *Atmos. Chem. Phys*. **2010**, *10*, 10923–10938, https://doi.org/10.5194/acp-10-10923-2010.

26. Macdonald, K. M.; Sharma, S.; Toom, D.; Chivulescu, A.; Platt, A.; Elsasser, M.; Huang, L.; Leaitch, R.; Chellman, N.; McConnell, J. R.; Bozem, H.; Kunkel, D.; Lei, Y. D.; Jeong, C.-H.; Abbatt,

J. P. D.; Evans, G. J. Temporally delineated sources of major chemical species in high Arctic snow, *Atmos. Chem. Phys*. **2018**, *18*, 3485–3503, https://doi.org/10.5194/acp-18-3485-2018.

27. von Schneidemesser, E.; Schauer, J. J.; Hagler, G. S.W.; Bergin, M. H. Concentrations and sources of carbonaceous aerosol in the atmosphere of Summit, Greenland. *Atmos. Environ.* **2009**, *43* (27), 4155-4162, DOI: org/10.1016/j.atmosenv.2009.05.043.

28. Pisso, I.; Sollum, E.; Grythe, H.; Kristiansen, N. I.; Cassiani, M.; Eckhardt, S.; Arnold, D.; Morton, D.; Thompson, R. L.; Groot Zwaaftink, C. D.; Evangeliou, N.; Sodemann, H.; Haimberger, L.; Henne, S.; Brunner, D.; Burkhart, J. F.; Fouilloux, A.; Brioude, J.; Philipp, A.; Seibert, P.; Stohl, A. The Lagrangian particle dispersion model FLEXPART version 10.4. *Geosci. Model Dev*. **2019**, *12*, 4955–4997, https://doi.org/10.5194/gmd-12-4955-2019.

29. Stohl, A.; Klimont, Z.; Eckhardt, S.; Kupiainen, K.; Shevchenko, V. P.; Kopeikin, V. M.; Novigatsky, A. N. Black carbon in the Arctic: the underestimated role of gas flaring and residential combustion emissions. *Atmos. Chem. Phys*. **2013**, *13,* 8833–8855; DOI 10.5194/acp-13-8833-2013.

30. Shevchenko, V. P.; Kopeikin V. M.; Evangeliou N.; Lisitzin, A. P.; Novigatsky, A. N.; Pankratova, N. V.; Starodymova, D. P.; Stohl, A.; Thompson, R. Atmospheric black carbon over the North Atlantic and the Russian Arctic Seas in summer-autumn time. Chemistry for Sustainable Development, **2016**, *24*, 441–446; DOI:10.15372/KhUR20160402.

31. Popovicheva, O. B.; Evangeliou, N.; Eleftheriadis, K.; Kalogridis, A. C.; Sitnikov, N.; Eckhardt, S.; Stohl, A. Black carbon sources constrained by observations in the Russian high Arctic. *Environ. Sci. Tech*. **2017**, *51*, 3871–3879, DOI: 10.1021/acs.est.6b05832.

32. Evangeliou, N.; Shevchenko, V. P.; Yttri, K. E.; Eckhardt, S.; Sollum, E.; Pokrovsky, O. S.; Kobelev, V. O.; Korobov, V. B.; Lobanov, A. A.; Starodymova, D. P.; Vorobiev, S. N.; Thompson, R. L.; Stohl, A. Origin of elemental carbon in snow from western Siberia and northwestern European Russia during winter–spring 2014, 2015 and 2016, *Atmos. Chem. Phys.* **2018**, *18*, 963–977, https://doi.org/10.5194/acp-18-963-2018.

33. Winiger, P.; Andersson, A.; Eckhardt, S.; Stohl, A.; Gustafsson, Ö. The sources of atmospheric black carbon at a European gateway to the Arctic. *Nat. Commun*. **2016**, *7*, 12776.

34. Winiger, P, Andersson, A., Eckhardt, S., Stohl, A., Semiletov, I. P., Dudarev, O. V., Charkin, A, Shakhova, N., Klimont, Z., Heyes, C., and Gustafsson, Ö.: Siberian Arctic black carbon sources constrained by model and observation, *Proc. Nat. Sci. Acad. USA*, **2017**, *114*, E1054–E1061, doi: 10.1073/pnas.1613401114.

35. Winiger, P.; Barrett, T. E.; Sheesley, R. J.; Huang, L.; Sharma, S.; Barrie, L. A.; Yttri, K. E.; Evangeliou, N.; Eckhardt, S.; Stohl, A.; Klimont, Z.; Heyes, C.; Semiletov, I. P.; Dudarev, O. V.; Charkin, A.; Shakhova, N.; Holmstrand, H.; Andersson, A.; Gustafsson, Ö. Source apportionment of

circum-Arctic atmospheric black carbon from isotopes and modeling. *Sci. Adv.* **2019**, *5*: eaau8052, doi: 10.1126/sciadv.aau8052.

36. Sinha, P. R.; Kondo, Y.; Goto-Azuma, K.; Tsukagawa, Y.; Fukuda, K.; Koike, M.; Ohata, S.; Moteki, N.; Mori, T.; Oshima, N.; Førland, E. J.; Irwin, M.; Gallet, J.-C.; Pedersen, C. A. Seasonal progression of the deposition of black carbon by snowfall at Ny-Ålesund, Spitsbergen. J. Geophys. Res.: Atmos., **2018**, *123*, 997–1016. https://doi.org/10.1002/2017JD028027.

37. Huang, K.; Fu, J. S.; Prikhodko, V. Y.; Storey, J. M.; Romanov, A.; Hodson, E. L.; Cresko, J.; Morozova, I.; Ignatieva, Y.; Cabaniss, J. Russian anthropogenic black carbon: Emission reconstruction and Arctic black carbon simulation. *J. Geophys. Res. – Atmos*. **2015**, *120*, 11,306–11,333; DOI 10.1002/2015JD023358.

38. Lamarque, J.-F.; Bond, T. C.; Eyring, V.; Granier, C.; Heil, A.; Klimont, Z.; Lee, D.; Liousse, C.; Mieville, A.; Owen, B.; Schultz, M. G.; Shindell, D.; Smith, S. J.; Stehfest, E.; Van Aardenne, J.; Cooper, O. R.; Kainuma, M.; Mahowald, N.; McConnell, J. R.; Naik, V.; Riahi, K.; van Vuuren, D. P. Historical (1850–2000) gridded anthropogenic and biomass burning emissions of reactive gases and aerosols: methodology and application, *Atmos. Chem. Phys*. **2010**, *10*, 7017–7039, doi:10.5194/acp-10-7017-2010.

39. van Marle, M. J. E.; Kloster, S.; Magi, B. I.; Marlon, J. R.; Daniau, A. L.; Field, R. D.; Arneth, A.; Forrest, M.; Hantson, S.; Kehrwald, N. M.; Knorr, W.; Lasslop, G.; Li, F.; Mangeon, S.; Yue, C.; Kaiser, J. W.; van der Werf, G. R.: Historic global biomass burning emissions for CMIP6 (BB4CMIP) based on merging satellite observations with proxies and fire models (1750-2015). *Geosci. Model Dev.* **2017**, *10*, 3329–3357, 10.5194/gmd-10-3329-2017.

40. Louchouarn, P.; Chillrud, S. N.; Houel, S.; Yan, B.; Chaky, D.; Rumpel, C.; Largeau, C.; Bardoux, G.; Walsch, D.; Bopp, R. F. Elemental and molecular evidence of soot- and char-derived black carbon inputs to New York City's atmosphere during the 20th century. *Environ. Sci. Technol*. **2007**, *41*, 82–87.

41. Nguyen, T. H.; Brown, R. A.; Ball, W. P. An evaluation of thermal resistance as a measure of black carbon content in diesel soot, wood char, and sediment. *Org. Geochem*. **2004**, *35*, 217–234.

42. Hoesly, R. M.; Smith, S. J.; Feng, L. Y.; Klimont, Z.; Janssens-Maenhout, G.; Pitkanen, T.; Seibert, J. J.; Vu, L.; Andres, R. J.; Bolt, R. M.; Bond, T. C.; Dawidowski, L.; Kholod, N.; Kurokawa, J.; Li, M.; Liu, L.; Lu, Z. F.; Moura, M. C. P.; O'Rourke, P. R.; Zhang, Q. Historical (1750–2014) anthropogenic emissions of reactive gases and aerosols from the Community Emissions Data System (CEDS), *Geosci. Model Dev.* **2018**, *11*, 369–408, 10.5194/gmd-11-369-2018.
